# Lifestyle eHealth and mHealth Interventions for Children and Adolescents: Systematic Umbrella Review and Meta–Meta-Analysis

**DOI:** 10.2196/69065

**Published:** 2025-10-17

**Authors:** Ben Singh, Mavra Ahmed, Amanda E Staiano, Maria F Vasiloglou, Claire Gough, Jasmine M Petersen, Zenong Yin, Corneel Vandelanotte, Chelsea Kracht, Janis Fiedler, Irina Timm, Joan Dallinga, Bridve Sivakumar, Hannes Baumann, Christopher Huong, Kathrin Wunsch, Mónica Suárez-Reyes, Stephanie Schoeppe, Alyssa M Button, Katherine Spring, Carol Maher

**Affiliations:** 1 Alliance for Research in Exercise Nutrition and Activity (ARENA) University of South Australia Adelaide Australia; 2 Department of Nutritional Sciences and Joannah and Brian Lawson Centre for Child Nutrition University of Toronto Toronto, ON Canada; 3 Pennington Biomedical Research Center Louisiana State University Baton Rouge, LA United States; 4 Nestlé Institute of Health Science Nestlé Research Lausanne Switzerland; 5 College of Nursing and Health Sciences Flinders University Adelaide Australia; 6 Department of Public Health University of Texas at San Antonio San Antonio, TX United States; 7 Physical Activity Research Group Appleton Institute Central Queensland University Rockhampton Australia; 8 University of Kansas Medical Centre Kansas City, KS United States; 9 Institute of Sports and Sports Science Karlsruhe Institute of Technology Karlsruhe Germany; 10 Centre of Expertise Health Innovation The Hague University of Applied Sciences The Hague The Netherlands; 11 Faculty of Health Science Ontario Tech University Ontario, ON Canada; 12 Institute for Movement Therapy and Movement-oriented Prevention and Rehabilitation German Sports University Cologne Cologne Germany; 13 Fresenius University of Applied Sciences Heidelberg Germany; 14 Escuela de Ciencias de la Actividad Física el Deporte y la Salud Universidad de Santiago de Chile Santiago Chile; 15 Virginia Commonwealth University Richmond, VA United States

**Keywords:** eHealth, mobile health, mHealth, lifestyle, physical activity, diet, sleep, sedentary behavior, digital interventions, health technologies, health behaviors

## Abstract

**Background:**

eHealth and mobile health (mHealth) interventions are promising in promoting healthy behaviors among children and adolescents.

**Objective:**

This systematic umbrella review and meta–meta-analysis aimed to evaluate the effectiveness of eHealth and mHealth interventions in promoting healthy behaviors among children and adolescents.

**Methods:**

Nine databases were searched for systematic reviews and meta-analyses of randomized controlled trials of eHealth and mHealth interventions targeting physical activity, sedentary behavior, sleep, and dietary outcomes in children and adolescents aged below 18 years. Screening, data extraction, and all assessments were completed by 2 reviewers. Study quality was assessed using the AMSTAR-2 (A Measurement Tool to Assess Systematic Reviews-2) checklist, and meta-analyses were conducted to combine effect sizes using random effects models. Subgroup analyses examined participant and intervention characteristics.

**Results:**

A total of 25 systematic reviews comprising 440 randomized controlled trials and 133,501 participants, mostly involving healthy children and adolescents (n=18, 72%) or those who were overweight or with obesity (n=4, 16%), were included. Interventions mostly included active video games or serious games (n=8, 32%), various mHealth interventions (n=6, 24%), various eHealth interventions (n=5, 20%), combined eHealth and mHealth interventions (n=4, 16%), wearables (n=1, 4%), and computer-based interventions (n=1, 4%). Most studies (n=18, 72%) had critically low AMSTAR-2 scores. Meta-analyses based on standardized mean difference (SMD) showed significant effects (all *P*<.05) for moderate to vigorous physical activity (SMD 0.18, 95% CI 0.09-0.27), total physical activity (SMD 0.24, 95% CI 0.13-0.35), fat intake (SMD 0.10, 95% CI 0.02-0.18), fruit and vegetable intake (SMD 0.11, 95% CI 0.00-0.22), BMI (SMD 0.19, 95% CI 0.11-0.27), and body weight (SMD 0.15, 95% CI 0.01-0.30). No effects were found for sedentary behavior (SMD 0.12, 95% CI –0.11 to 0.35) or sleep (SMD 0.27, 95% CI –0.09 to 0.63). Shorter interventions (lasting <8 weeks) had a greater effect on moderate to vigorous physical activity than longer interventions (lasting ≥8 weeks), while longer interventions (lasting ≥12 weeks) had a greater effect on BMI compared with shorter interventions.

**Conclusions:**

eHealth and mHealth interventions offer modest but meaningful improvements in physical activity, diet, and weight management in children and adolescents, with important implications for integrating digital tools into health promotion strategies.

**Trial Registration:**

PROSPERO CRD42024537019; https://www.crd.york.ac.uk/PROSPERO/view/CRD42024537019

## Introduction

### Background

In recent years, digital health interventions, particularly eHealth and mobile health (mHealth) platforms, have shown promise in promoting healthy behaviors [1]. eHealth refers to the use of digital technologies, such as websites and online platforms, to deliver health services and information, while mHealth specifically refers to mobile-based health technologies, such as smartphone apps, SMS text messages, and wearable devices [1]. These interventions use digital tools, such as smartphones, apps, wearable activity trackers, and websites, to deliver health-related information, social support, engagement strategies, and behavior change programs [1]. Key target behaviors for health promotion in children and adolescents include physical activity, sedentary behavior, sleep, and healthy eating. These behaviors are considered critical for overall health and chronic disease prevention, as they are modifiable risk factors with a substantial impact on long-term health outcomes [2-5]. Higher levels of physical activity, especially moderate to vigorous physical activity (MVPA), are associated with a reduced risk of chronic diseases, such as cardiovascular disease and type 2 diabetes [6,7]. In contrast, high levels of sedentary behavior are linked to obesity and metabolic syndrome [8,9]. Adequate sleep in both quality and duration is vital for cognitive function and mental health [10], while a balanced diet is essential for maintaining a healthy weight and supporting optimal growth and development [11]. It is important to recognize that, in addition to physical activity, sedentary behavior, sleep, and healthy eating, other lifestyle factors—such as stress management, social engagement, and substance use—also play significant roles in overall health. In addition, weight-related outcomes, such as BMI, are commonly used indicators of lifestyle-related health status as they reflect the cumulative impact of multiple health behaviors [12]. By targeting these behaviors early, eHealth and mHealth interventions offer valuable opportunities to establish lifelong healthy habits, which can have a profound impact on reducing the risk of chronic diseases and improving overall well-being in the long term.

Despite known benefits, nonadherence to recommended levels of physical activity, sedentary behavior, sleep, and healthy eating remains prevalent among children and adolescents [13,14], highlighting the need for cost-effective and scalable interventions. Widespread internet access has enabled the use of eHealth and mHealth interventions that use behavior change techniques, such as goal setting, self-monitoring, feedback, and social support [1]. They also leverage gamification, tailored messaging, and machine learning to enhance engagement [1]. Addressing health behaviors in children and adolescents is imperative due to the significant impact on their immediate well-being and long-term health outcomes [15]. Early intervention during these formative years can establish a foundation for a healthy lifespan [16]. Moreover, children and adolescents are particularly receptive to digital technology, rendering eHealth and mHealth interventions promising for this demographic [17].

The evidence base for children’s eHealth and mHealth interventions has evolved significantly. Early studies were often small, with lower quality designs, but the field has evolved to incorporate higher quality studies, particularly randomized controlled trials (RCTs), and statistically powered studies, which offer more robust evidence of effectiveness [18]. This progression has been accompanied by numerous systematic reviews, each with varying foci and inclusion criteria [19,20]. Despite the expansion of the evidence base, the sheer volume and heterogeneity of studies make it challenging to discern the overall effectiveness of these interventions. Umbrella review methodology offers the ability to consolidate evidence and clarify which eHealth and mHealth intervention approaches are effective and most promising. To date, there have been 2 umbrella reviews, but with a limited scope. Rodríguez-González et al [19] focused on app-based interventions, finding that apps can improve physical activity, reduce sedentary behavior, and enhance diet quality among children and adults. Prowse and Carsley [20], focusing on children’s nutrition, revealed small and inconsistent effects of digital interventions on overall dietary outcomes, with promising results limited to increasing fruit and vegetable intake. These reviews had narrow scopes, focusing exclusively on nutrition interventions [20] or solely on apps [19] or evaluating children and adults together [19]. Neither review quantitatively pooled results through meta-analysis [19,20].

### Objectives

A comprehensive umbrella review is needed to guide research, practice, and policy in this evolving field. This review aims to provide a thorough overview of the effectiveness of eHealth and mHealth interventions on physical activity, sedentary behavior, sleep, and dietary outcomes in children and adolescents, using high-quality research designs and quantitative meta-analysis. These 4 outcomes (physical activity, sedentary behavior, sleep, and diet) were selected because they are critical, modifiable behaviors that significantly influence both immediate and long-term health, particularly in children and adolescents [13-15]. Subgroup analyses focus on age, sex, population type, intervention type, and study quality. Our review offers a comprehensive analysis across various subgroups, addressing gaps in the existing literature and providing insights to inform future research and clinical practice.

## Methods

### Protocol and Registration

The protocol was preregistered on PROSPERO (CRD42024537019) and is reported in accordance with PRISMA (Preferred Reporting Items for Systematic Reviews and Meta-Analyses) guidelines (Multimedia Appendix 1) [21].

### Selection Criteria

The inclusion and exclusion criteria were formulated using the participants, intervention, comparison, outcomes, and study design framework, as shown in Textbox 1 [22].

**Inclusion and exclusion criteria.**

**Inclusion criteria**

- Population: reviews involving children and adolescents aged below 18 years with or without health conditions were included. Studies including adults were eligible if the meta-analysis results for children and adolescents were reported separately.
- Intervention: reviews were included if more than 75% of the studies evaluated eHealth or mobile health interventions targeting physical activity, sedentary behavior (eg, screen time, leisure screen time, and sitting time), sleep (eg, sleep quality and sleep duration), or diet (eg, fruit and vegetable intake and dietary quality). Reviews involving noneligible interventions were included if they reported separate results for eligible interventions.
- Comparators: systematic reviews and meta-analyses were eligible if at least 75% of the included trials compared eligible interventions to no intervention, usual care, a sham intervention, or an attention control that did not involve improving physical activity, sleep, or diet or reducing sedentary behavior.
- Outcomes: main outcomes were changes in physical activity, sedentary behavior, sleep, or diet. Secondary outcomes included BMI, body weight, waist circumference, and body fat.
- Study design: systematic reviews, including meta-analysis results of at least 1 outcome of interest, were eligible. Systematic reviews were eligible if they included at least 75% randomized controlled trials (RCTs) or if they reported separate meta-analysis results for RCTs only.

**Exclusion criteria**

- Intervention: reviews with more than 25% ineligible interventions were excluded.
- Study design: narrative reviews, scoping reviews, and systematic reviews without meta-analyses or with non-RCTs were excluded.

### Search Strategy

Nine databases (CINAHL, Cochrane Library, Embase via OVID, MEDLINE via OVID, Emcare via OVID, ProQuest Central, ProQuest Nursing and Allied Health Source, PsycINFO, and Scopus) were systematically searched using subject headings, keywords, and Medical Subject Headings (MeSH) terms related to “eHealth,” “mHealth,” “physical activity,” “sedentary behaviour,” “sleep,” “diet,” “children and adolescents,” and “systematic review” (refer to Multimedia Appendix 2 for the full search strategy). The database searches were limited to peer-reviewed journal articles published in the English language until April 6, 2024.

### Data Management and Extraction

Search results were imported into EndNote (Version 20; Clarivate), and duplicates were removed. Results were then exported to Covidence (Veritas Health Innovation Ltd), and title and abstract screening, full-text screening, data extraction, and study quality scoring were performed. All screening, data extraction, and study quality assessments were completed independently in duplicate by 2 reviewers (BS, MA, AES, MFV, CG, JMP, ZY, CV, CK, JF, IT, JD, BS, HB, CH, KW, MS-R, SS, AMB, KS, and CM), and discrepancies were resolved by a third reviewer. A standardized Covidence data extraction form was used to extract information on study characteristics, population characteristics, intervention characteristics, outcomes of interest, and results.

Study quality of the included reviews was evaluated using the AMSTAR-2 (A Measurement Tool to Assess Systematic Reviews-2) checklist [23] by 2 reviewers (BS, MA, AES, MFV, CG, JMP, ZY, CV, CK, JF, IT, JD, BS, HB, CH, KW, MS-R, SS, AMB, KS, and CM), and discrepancies were resolved by a third reviewer. This tool involves 16 items that are scored as either *yes, partial yes,* or *no.* Seven of the AMSTAR-2 items are considered *critical,* and 9 items are *noncritical* [23]. The *critical* items include protocol registration, search strategy, study exclusions, study quality assessment, meta-analysis methods, study quality interpretation, and publication bias. Reviews were scored as either *critically low confidence* (>1 critical weakness and ≥3 noncritical weaknesses), *low confidence* (>1 critical weakness and <3 noncritical weaknesses), *moderate confidence* (1 critical weakness and <3 noncritical weaknesses), or *high confidence* (no critical weakness and <3 noncritical weaknesses) [23].

### Umbrella Review Synthesis Methods

The assessment of overlap between the studies included in the reviews was carried out using the corrected covered area (CCA) [24]. The CCA was calculated using the following equation:

CCA = (N – r) ÷ (r × c) – r

where N represents the total number of RCTs across all reviews (including duplicates), r is the number of unique RCTs (excluding duplicates), and c is the number of reviews included in the analysis [24]. The categories used to classify the overlap were 0% to 5% (slight), 6% to 10% (moderate), 11% to 15% (high), and more than 15% (very high overlap) [25].

Meta-analyses of the outcomes of interest were conducted by pooling the effect sizes and 95% CIs reported in each meta-analysis, using a random-effects model for outcomes that were reported in at least 2 studies. The results of all meta-analyses were presented visually using forest plots. Separate meta-analyses were performed for standardized effect sizes (eg, standardized mean difference [SMD]) and unstandardized effect sizes (eg, mean difference [MD]). The meta-analyzed effect sizes (SMDs or MDs) were reported with their corresponding 95% CIs. For meta-analyses of SMD, positive effect sizes indicate that the effects favor the intervention. Subgroup analyses were carried out for age (individuals aged <13 years or ≥13 years), sex (female, male, or not reported), population (general population and chronic disease), intervention type (mobile apps, web based, SMS text messages, and mixed [which included combinations of at least 3 of the other modes]), and AMSTAR-2 rating (critically low, low, moderate, or high) if more than 1 eligible meta-analysis was included in at least 2 of the groups. The classification of eHealth and mHealth interventions was informed by established definitions and frameworks in the digital health literature, distinguishing eHealth as primarily web-based interventions and mHealth as mobile-based (eg, apps, SMS text messages, and wearable devices) [1]. This approach aligned with previous umbrella reviews and systematic reviews evaluating digital health tools for behavior change in youth populations [19,20]. The *I*^2^ statistic was used to quantify the proportion of the overall outcome variability [26], with the following values used to determine the level of heterogeneity: low heterogeneity: *I*^2^=0% to 25%; moderate heterogeneity: *I*^2^ ≥25% to 50%; and high heterogeneity: *I*^2^ ≥75% to 100% [27]. To evaluate potential publication bias, funnel plots were generated, and the presence of asymmetries or missing data sections was visually inspected for meta-analyses that included at least 10 studies [28]. The magnitude of effect was classified using the following criteria: small effect: less than 0.20, medium effect: 0.20 to 0.50, and large effect: greater than 0.50 [29]. A *P* value of <.05 was considered statistically significant. All meta-analyses were performed using Stata (version 16, StataCorp) software.

Levels of evidence and grades for recommendations [30] were used to classify the overall level of evidence as grade A—consistent level 1 studies (ie, systematic reviews of RCTs or individual RCTs), grade B—consistent level 2 (ie, systematic reviews of cohort studies or individual cohort studies) or 3 studies (ie, systematic reviews of case-control studies or individual case-control studies) or extrapolations from level 1 studies, grade C—level 4 studies (ie, case series) or extrapolations from level 2 or 3 studies, or grade D—level 5 evidence (ie, expert opinion without explicit critical appraisal) or troublingly inconsistent or inconclusive studies of any level [30].

## Results

### Overview

**Figure 1:**
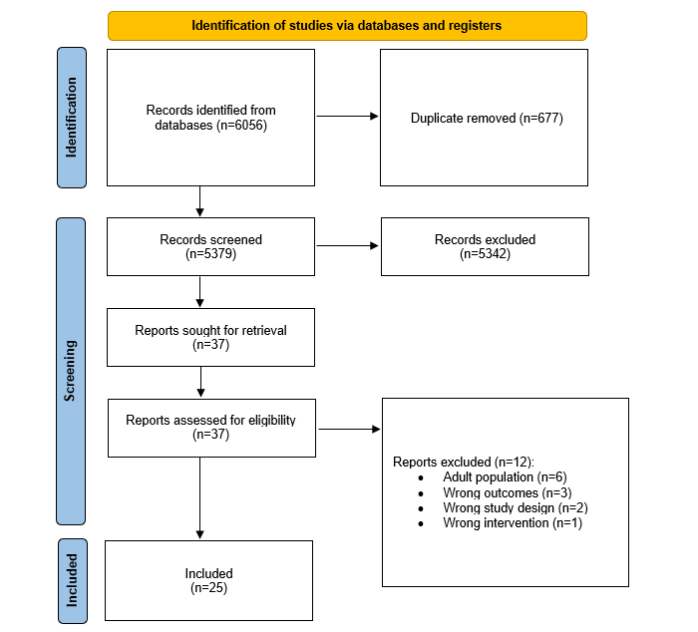
PRISMA (Preferred Reporting Items for Systematic Reviews and Meta-Analyses) flowchart.

The database search identified 6056 results. After screening, 25 (0.4%) systematic reviews and meta-analyses met the eligibility criteria and were included in the analysis. The PRISMA flowchart, showing the reasons for exclusions, is presented in Figure 1. A list of reasons for excluding studies after the full-text review can be found in Multimedia Appendix 2 [31-67]. The 25 included reviews comprised 440 trials involving 133,501 participants. The overall CCA was 3.28%, indicating a slight overlap across the systematic reviews.

A summary of the participant demographics, such as age and population groups, as well as the characteristics of the interventions, is shown in Multimedia Appendix 2 [31-55]. Of the 25 included studies, mean participant age in most (n=23, 92%) systematic reviews ranged between 3 and 18 years, and all (n=25, 100%) reviews included both female and male participants. Most (n=8, 32%) studies in the systematic reviews involved healthy children and adolescents [31-48]), 4(16%) systematic reviews involved children and adolescents who were overweight or with obesity [49-52], 1 (4%) involved infants and children [53], 1 (4%) involved children with a chronic disease [54], and 1 (4%) involved children and young people living with juvenile idiopathic arthritis [55]. The interventions in the systematic reviews involved active video games or serious games (n=8, 32%) [35,39,41,45,49,51,52,54], various mHealth interventions (n=6, 24%) [31,34,36,38,46,48], various eHealth interventions (eg, websites and social media groups; n=5, 20%) [33,37,43,44,50], various eHealth and mHealth interventions combined (eg, mobile apps and SMS text message interventions; n=4, 16%) [40,42,53,55], wearables (n=1, 4%) [47], and computer-based interventions (n=1, 4%) [32]. The systematic reviews included interventions that targeted obesity and weight management (n=11, 44%) [34,35,38,41-43,47-51], physical activity levels (n=6, 24%) [31,36,39,44,46,54], multiple lifestyle behaviors or health promotion (n=3, 12%) [33,40,45], 24-hour movement behaviors (n=1, 4%) [37], diet (n=1, 4%) [32], sleep (n=1, 4%) [53], juvenile idiopathic arthritis management (n=1, 4%) [55], and health-related physical fitness and motor competence (n=1, 4%) [52].

The included reviews had either a moderate (2/25, 8%) [33,44], low (5/25, 20%) [31,32,37,39,50], or critically low (18/25, 72%) [34-36,38,40-43,45-49,51-55] AMSTAR-2 score (refer to Multimedia Appendix 2 [31-55] for full study quality scoring). Common limitations included not providing a list of full-text exclusions (22/25, 88%) and not describing the funding sources of the included studies (25/25, 100%).

### Results of the Meta-Analyses

#### Physical Activity

##### Overview

**Figure 2:**
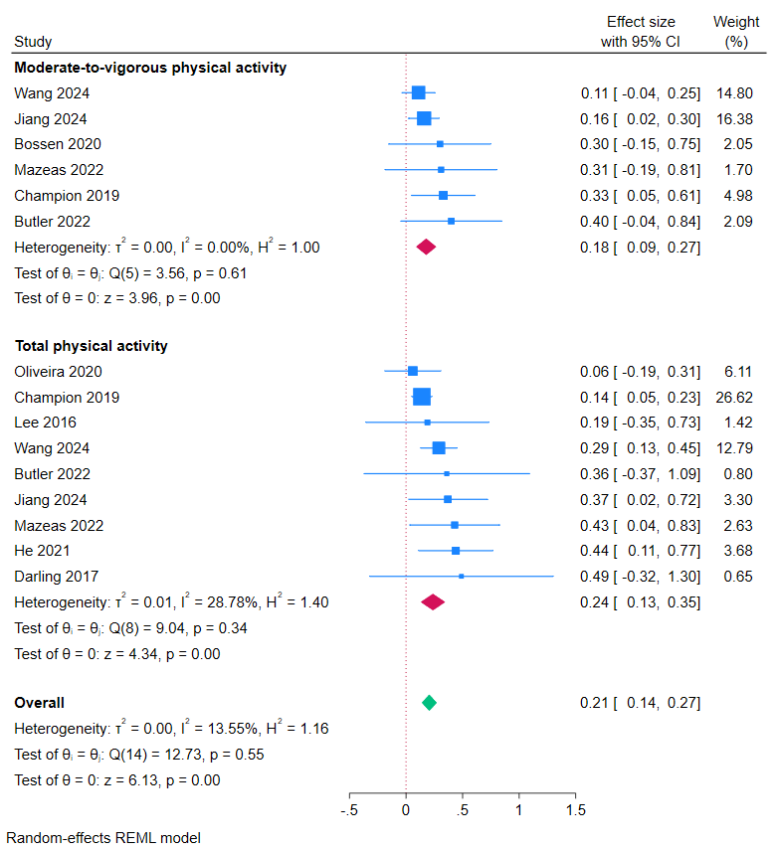
Meta-analysis of effects of eHealth and mobile health interventions on physical activity outcomes in children and adolescents based on standardized mean difference. Positive effect sizes favor intervention.

Results of meta-analyses based on SMD showed a significant effect in favor of eHealth and mHealth interventions on MVPA (SMD 0.18, 95% CI 0.09-0.27; *I*^2^=0%; *P*<.001; 6/25, 24%) and total physical activity (combined light, moderate, and vigorous; SMD 0.24, 95% CI 0.13-0.35; *I*^2^=28.8%; *P*<.001; 9/25, 36%; Figure 2 [33,37-39,41,46,55]). Results based on MD showed no significant effect on MVPA (MD 1.78 minutes/day, 95% CI –3.79 to 7.35; *I*^2^=55.4%; *P*=.53; 2/25, 8%; Multimedia Appendix 2 [40,44]; there were insufficient MD data for total physical activity).

##### Levels of Evidence and Grades for Recommendations

Evidence for improvements in MVPA and total physical activity was based on level 1 studies (systematic reviews of RCTs) with consistent effects. Due to the low methodological quality of many reviews, the grade of recommendation was grade B.

#### Diet

##### Overview

**Figure 3:**
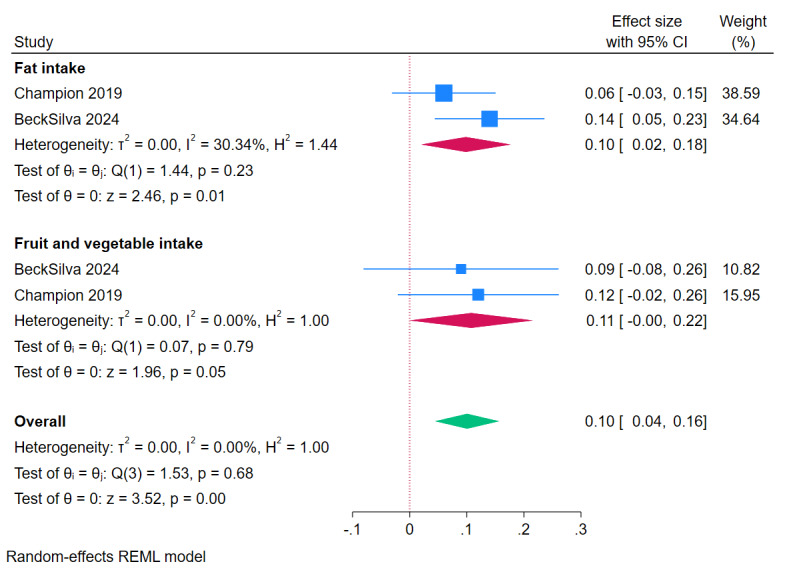
Meta-analysis of effects of eHealth and mobile health interventions on dietary outcomes in children and adolescents based on standardized mean difference. Positive effect sizes favor intervention.

Results of meta-analyses based on SMD showed a significant effect of eHealth and mHealth interventions on fat intake (SMD 0.10, 95% CI 0.02-0.18; *I*^2^=30.3%; *P*=.01; 2/25, 8%) and fruit and vegetable intake (SMD 0.11, 95% CI 0.00-0.22; *I*^2^=0%; *P*=.05; 2/25, 8%; Figure 3 [32,33]). There were insufficient MD data for diet outcomes.

##### Levels of Evidence and Grades for Recommendations

Improvements in fat, fruit, and vegetable intake were supported by level 1 evidence. However, the limited number of meta-analyses and low review quality supported a grade B recommendation.

#### Sedentary Behavior

##### Overview

**Figure 4:**
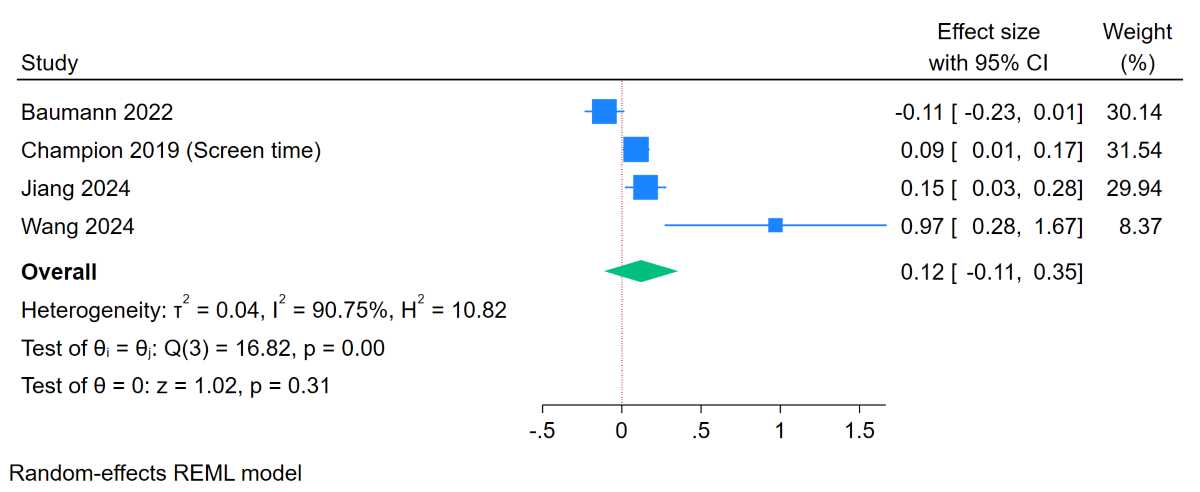
Meta-analysis of effects of eHealth and mobile health interventions on sedentary behavior in children and adolescents based on standardized mean difference. Positive effect sizes favor intervention.

Results of meta-analyses based on SMD showed no effect of eHealth and mHealth interventions on sedentary behavior (SMD 0.12, 95% CI –0.11 to 0.35; *I*^2^=90.8%; *P*=.31; 4/25, 16%; Figure 4 [31,33,37,46]). Meta-analyses based on MD showed significant reductions in overall sedentary behavior (MD 24.08 minutes/day, 95% CI –37.97 to –10.20; *I*^2^=0%, *P*<.001; 2/25, 8%; Multimedia Appendix 2 [40,44]) and screen time (MD 21.83 minutes/day, 95% CI –42.77 to –0.89; *I*^2^=0%; *P*=.04; 2/25, 8%; Multimedia Appendix 2 [40,44]).

##### Levels of Evidence and Grades for Recommendations

Mixed findings with high heterogeneity and inconsistent results across SMD and MD analyses resulted in lower confidence in this outcome. Despite being level 1 evidence, the recommendation was grade C.

#### Sleep

##### Overview

Results of meta-analyses based on SMD showed no effect of eHealth and mHealth interventions on sleep duration (SMD 0.27, 95% CI –0.09 to 0.63; *I*^2^=78.2%; *P*=.14; 2/25, 8%; Multimedia Appendix 2 [37,53]). There were insufficient MD data for sleep outcomes.

##### Levels of Evidence and Grades for Recommendations

Findings for sleep duration were nonsignificant and based on limited, heterogeneous data from low-quality reviews. This outcome was supported by level 1 evidence but was graded as grade C.

#### BMI, Body Weight, Body Fat, and Waist Circumference

##### Overview

**Figure 5:**
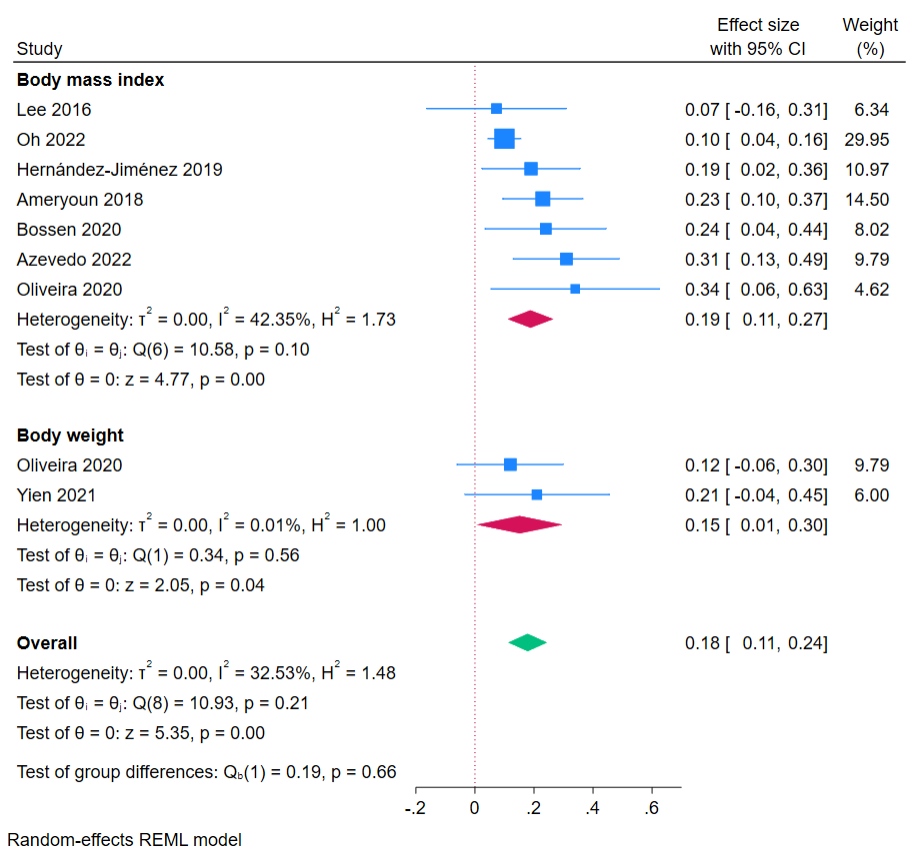
Meta-analysis of effects of eHealth and mobile health interventions on BMI and body weight in children and adolescents based on standardized mean difference. Positive effect sizes favor intervention.

Results of meta-analyses based on SMD showed a significant effect in favor of eHealth and mHealth interventions on BMI (SMD 0.19, 95% CI 0.11-0.27; *I*^2^=42.4%; *P*<.001; 7/25, 28%) and body weight (SMD 0.15, 95% CI 0.01-0.30; *I*^2^=0%; *P*=.04; 2/25, 8%; Figure 5 [35,38,40,41,48-50,54]). Meta-analysis results based on MD showed a significant reduction in BMI (MD –0.22 kg/m^2^, 95% CI –0.33 to –0.10; *I*^2^=36.99%; *P*<.001; 7, 28%; Multimedia Appendix 2 [32,42,43,46,47]) and body weight (MD –0.99 kg, 95% CI=–1.51 to –0.47; *I*^2^=0%; *P*<.001; 9/25, 36%; Multimedia Appendix 2 [43,47]). Results of meta-analyses based on MD also showed a significant reduction in body fat percentage (MD –0.47%, 95% CI –0.66 to –0.29; *I*^2^=0%; *P*<.001; 4/25, 16%; Multimedia Appendix 2 [40,43,47,52]) and no significant change in waist circumference (MD –0.15, 95% CI –0.87 to 0.57; *I*^2^=56.05%; *P*=.68; 4/25, 16%; Multimedia Appendix 2 [43,46,47,52]).

##### Levels of Evidence and Grades for Recommendations

BMI, body weight, and body fat showed consistent and significant improvements across level 1 studies. Due to moderate heterogeneity and review quality concerns, the recommendation was grade B. Waist circumference showed no effect.

### Subgroup Analyses

Subgroup analyses were conducted to explore whether intervention effectiveness varied according to age, intervention duration, intervention type, and study quality score, based on the availability of data from at least 2 eligible meta-analyses per subgroup.

#### Age

There were no significant subgroup effects for age (<13 or ≥13 years) on MVPA (Q_b_ (1)=0.02; *P*=.88; Multimedia Appendix 2), total physical activity (Q_b_ (1)=3.21; *P*=.38; Multimedia Appendix 2), BMI (Q_b_ (1)=0.35; *P*=.55; Multimedia Appendix 2), and sedentary behavior (Q_b_ (1)=2.25; *P*=.13; Multimedia Appendix 2).

#### Intervention Duration

Interventions that lasted less than 8 weeks had a greater effect on MVPA compared with those that lasted 8 weeks or longer (SMD 0.86 vs 0.19; Q_b_ (1)=9.03; *P*<.001; Multimedia Appendix 2). In contrast, longer interventions (≥12 weeks) had a greater effect on BMI compared with shorter interventions (SMD 0.46 vs –0.07; Q_b_ (1)=6.08; *P*<.001; Multimedia Appendix 2). There was no difference between shorter and longer interventions for total physical activity (Q_b_ (1)=1.66; *P*=.20; Multimedia Appendix 2) and sedentary behavior (Q_b_ (1)=2.25; *P*=.13; Multimedia Appendix 2).

#### Intervention Type

There were no significant subgroup effects for intervention type (eHealth only, mHealth only, exergames, eHealth and mHealth mixed, web based only, app only, SMS text message only, or SMS text message plus app) on MVPA (Q_b_ (7)=7.08; *P*=.42; Multimedia Appendix 2) and total physical activity (Q_b_ (3)=1.28; *P*=.73; Multimedia Appendix 2).

Wearable-only interventions showed larger effects on sedentary behavior (SMD 0.97) compared to various eHealth (SMD 0.11) and mHealth (SMD –0.11) interventions (Q_b_ (2)=16.2; *P*<.001; Multimedia Appendix 2).

App-only interventions (SMD 0.78) showed larger effects compared to various eHealth and mHealth interventions, exergames, and web-based only interventions on BMI (SMD range 0.00-0.31; Q_b_ (5)=38.00; *P*<.001; Multimedia Appendix 2).

#### Study Quality Score

There were no significant subgroup effects for study quality score (moderate, low, or critically low on MVPA; Q_b_ (2)=1.06; *P*=.59; Multimedia Appendix 2), total physical activity (Q_b_ (2)=4.48; *P*=.11; Multimedia Appendix 2), and BMI (Q_b_ (1)=2.09; *P*=.15; Multimedia Appendix 2).

Studies rated as critically low (ie, highest risk of bias) showed the greatest effects on sedentary behavior (SMD 0.97) compared with low- (SMD 0.02) and moderate-rated studies (SMD 0.09; Q_b_ (2)=6.44; *P*=.04; Multimedia Appendix 2).

### Publication Bias

A visual examination of funnel plots for physical activity outcomes (Multimedia Appendix 2) revealed some asymmetry, with a gap in the bottom left quadrant. This suggested a potential lack of smaller studies reporting negative effect sizes. The estimated true effect size (Cohen *d* or θ) was –0.17. For other outcomes, there was an insufficient number of systematic reviews (<10) to create funnel plots.

## Discussion

### Principal Findings

This comprehensive umbrella review synthesized evidence from 25 systematic reviews and meta-analyses, encompassing 440 trials involving 133,501 participants, to evaluate the effectiveness of eHealth and mHealth interventions for promoting healthy lifestyle behaviors in children and adolescents. eHealth and mHealth interventions demonstrated small but positive effects on increasing MVPA (SMD 0.18) and total physical activity (SMD 0.24); improving dietary habits, including fat intake (SMD –0.10) and fruit and vegetable consumption (SMD 0.11); and reducing BMI (SMD –0.19; MD –0.22 kg/m^2^) and body weight (SMD –0.15; MD –0.99 kg). While SMD showed no significant effect on sedentary behavior, MDs indicated reductions in overall sedentary time (–24 minutes/day) and screen time (–22 minutes/day). However, the interventions did not significantly impact sleep duration. Although the effect sizes for health behaviors were small, even modest improvements in physical activity, sedentary behavior, and diet can contribute to significant long-term health benefits, particularly when sustained over time. These small changes are particularly important during childhood and adolescence when healthy habits are formed and can have a lasting impact on future health [15,16]. Moreover, the scalability of digital interventions means that even small individual effects can translate into substantial population-level impact when delivered to large numbers of people. These findings suggest that digital health interventions can play a valuable role in promoting healthier lifestyles among youth, although with varying degrees of effectiveness across different health behaviors.

Our findings demonstrate significant improvements in physical activity, dietary behaviors, and weight-related outcomes following eHealth and mHealth interventions. While the observed effect sizes were generally small, they are comparable or even favorable compared to those found in traditional face-to-face interventions targeting health behaviors in children and adolescents. For instance, our study showed more favorable effects on BMI (SMD –0.19) and body weight (SMD –0.15) compared to the meta-analysis by Guerra et al [68] of 12 school-based physical activity interventions (BMI: SMD –0.02; body weight: SMD –0.07). Similarly, our results for MVPA were more promising than those reported in the meta-analysis by Love et al [69] of 17 school-based physical activity interventions. Regarding dietary outcomes, our findings for fruit and vegetable consumption (SMD 0.11) were comparable to those reported in school-based nutrition studies (SMD 0.23) [70], although slightly smaller. This difference might be attributed to the ability of school-based interventions to directly supervise or provide fruit and vegetable consumption, which is not feasible with eHealth and mHealth approaches. Overall, these comparisons suggest that digital health interventions can be as effective, if not more so, than traditional intervention methods.

Our analysis revealed mixed results for sedentary behavior and sleep outcomes. While SMD effects showed no significant effect on overall sedentary behavior, MD effects indicated significant reductions in sedentary behavior (24 minutes/day) and screen time (22 minutes/day) outcomes. This discrepancy might be due to measurement heterogeneity across studies or the challenge of capturing nuanced changes in sedentary patterns. Regarding sleep, our review found no statistically significant impact on sleep duration; however, this conclusion is based on limited data from a single meta-analysis and should therefore be interpreted with caution. This highlights a critical gap in the current research landscape, as optimal sleep is crucial for children’s growth, cognitive development, and mental health [71]. The lack of significant effects on sleep and the mixed results for sedentary behavior underscore the need for more targeted, high-quality research in these areas.

Our subgroup analyses provided valuable insights regarding factors that may optimize intervention effects. Wearable-only interventions demonstrated substantially larger effects on reducing sedentary behavior (SMD 0.97) compared to various eHealth (SMD 0.11) and mHealth (SMD –0.11) interventions. This finding suggests that wearable devices may be particularly effective in promoting movement and reducing sedentary time among children and adolescents. Furthermore, intervention duration emerged as a significant moderator of outcomes. Shorter interventions (<8 weeks) demonstrated larger effects for increasing physical activity, while longer durations (≥12 weeks) were more effective for reducing BMI. This pattern aligns with previous research, demonstrating that people’s motivation often declines during longer physical activity interventions, highlighting the need for strategies to sustain long-term engagement [72]. In contrast, the greater effectiveness of longer interventions for BMI reduction is consistent with the understanding that sustained caloric deficits and lifestyle changes are necessary for meaningful weight management [73]. Regarding intervention type, app-based interventions showed the most promising results for improving BMI compared to other digital formats. This may be attributed to the unique capabilities of apps, such as frequent self-monitoring, goal tracking, tailored feedback, and gamification elements, which have been shown to be effective behavior change techniques for successful weight loss intervention [74-76]. These findings suggest that tailoring intervention duration to specific health outcomes and leveraging the interactive features of mobile apps could enhance the impact of digital health initiatives for children and adolescents. Although no significant subgroup effects were found for age, it is important to acknowledge that developmental differences between children and adolescents may influence how interventions are designed, delivered, and received. Factors such as cognitive maturity, autonomy, and technology use habits vary across age groups and may affect engagement and outcomes. The lack of age effects in our analyses may reflect limited power in subgroup comparisons or insufficient age-specific tailoring in many interventions. Future research should explore age-tailored strategies to enhance relevance and effectiveness. Due to the inconsistency in reporting adherence and engagement across studies, we were unable to conduct a comprehensive analysis of their role in this review. Future research should investigate adherence and engagement as key mediators of the effectiveness of eHealth and mHealth interventions. A better understanding of how these factors influence behavior change could help optimize interventions and enhance their real-world applicability. Furthermore, to ensure long-term health benefits, future eHealth and mHealth interventions should incorporate strategies to maintain participant engagement, such as regular progress tracking, personalized feedback, gamification, and social support features. These strategies can help individuals sustain healthy behaviors over time and prevent dropout.

This umbrella review synthesizes evidence from 25 systematic reviews and meta-analyses, comprising 440 trials with 133,501 participants, providing a comprehensive assessment of eHealth and mHealth interventions for children and adolescents. Adhering to PRISMA guidelines ensures transparency and reproducibility, and using rigorous screening and extraction processes in duplicate minimizes study overlap (CCA=3.28%), enhancing reliability and focus on digital health interventions for youth.

### Limitations

This umbrella review has several limitations, primarily related to the varying methodological quality of included reviews. Many (23/25, 92%) studies were rated as low or critically low confidence per AMSTAR-2 criteria, often due to inconsistent reporting, lack of preregistration, and limited assessments of bias and heterogeneity. These quality issues may reduce confidence in the reported effects, particularly for physical activity outcomes, and suggest that some effects could be overestimated due to potential publication bias favoring positive results. Future research should improve rigor by adopting standardized protocols, thorough bias evaluations, and consistent subgroup analyses. In addition, insufficient data limited subgroup analyses across key populations and intervention characteristics, preventing a detailed understanding of intervention effectiveness across diverse groups and settings. Furthermore, the type of outcome measurement (objective vs self-reported) and the identity of the respondent (eg, child, adolescent, or parent) were not consistently reported across reviews, limiting our ability to explore these factors in subgroup analyses. More granular data reporting in future studies would enable more tailored and reliable insights for various demographic contexts. Although many studies included in this review had low-quality ratings, our subgroup analyses indicate that these studies had minimal impact on the overall findings for most outcomes, except for sedentary behavior. This suggests that while study quality is a critical factor, the trends identified in the meta-analyses remain relevant. It is important to note that the strict criteria of the AMSTAR 2 tool, particularly for items such as comprehensive search strategies, meant that even well-conducted reviews often did not meet all requirements for a full “yes” rating, potentially underestimating their methodological quality.

The findings highlight significant clinical implications for child and adolescent health stakeholders. eHealth and mHealth interventions show promising but modest improvements in physical activity, dietary habits, and BMI. Stakeholders, including health care providers and educators, should consider integrating these digital tools, particularly app-based platforms, into youth health promotion strategies. Tailoring intervention durations to specific outcomes, such as shorter durations for increasing physical activity and longer durations for reducing BMI, is recommended. Early intervention is crucial, with younger children showing greater reductions in sedentary behavior compared to adolescents, suggesting potential roles for schools and community organizations in integrating these interventions into existing programs.

In the rapidly evolving digital health landscape, curated app libraries and systematic frameworks are urgently needed to assist professionals in selecting effective behavior change apps confidently. Public health agencies can contribute by developing guidelines and resources for evidence-based digital health tools. Future research should focus on improving the effectiveness of eHealth and mHealth interventions for children and adolescents by conducting more high-quality primary RCTs, exploring innovative strategies for sustaining long-term engagement, assessing long-term intervention effects, investigating digital interventions for sleep and sedentary behavior, and optimizing integration with other health promotion strategies. These results have important implications for research, practice, and policy in the field of digital health interventions for youth. Researchers should focus on further investigating the specific features of wearable devices and mobile apps that contribute to their effectiveness in reducing sedentary behavior and BMI, respectively. This could involve systematic evaluations of intervention components and exploring combinations of these promising approaches with other digital tools. In practice, health care providers and educators should consider prioritizing the integration of evidence-based wearable devices and mobile apps into their health promotion strategies for children and adolescents, particularly when targeting sedentary behavior and weight management. Policymakers should take note of these findings when developing guidelines for digital health interventions and allocating resources for their implementation in various settings, including schools and community organizations. Future research should also explore how to enhance the long-term effectiveness of these interventions, possibly by combining the most effective digital approaches with traditional face-to-face components to create comprehensive, multilevel interventions that address various determinants of health behaviors in youth.

### Conclusions

In conclusion, this umbrella review shows that eHealth and mHealth interventions offer significant benefits for improving physical activity, diet quality, and weight management in children and adolescents. While effects on sedentary behavior were mixed and sleep outcomes showed no significant improvement, digital technologies have potential for promoting healthier lifestyles among youth, proving comparable or superior to traditional interventions in some aspects. On the basis of our findings of small but positive effects across these domains, we suggest that future research should focus on how best to translate these evidence-based interventions into real-world settings at scale. Particularly, studies exploring implementation strategies; equity of access; and integration into school, primary care, or community settings would be valuable. In addition, while our review did not assess engagement or adherence directly, future umbrella or systematic reviews focusing specifically on these factors may help complement and extend our findings.

## Appendix

Multimedia Appendix 1 PRISMA checklist.

Multimedia Appendix 2 Search details, exclusions, included studies, and meta-analyses of eHealth/mHealth effects on youth health outcomes.

## Abbreviations

AMSTAR-2: A Measurement Tool to Assess Systematic Reviews-2
CCA: corrected covered area
MD: mean difference
MeSH: Medical Subject Headings
mHealth: mobile health
MVPA: moderate to vigorous physical activity
PRISMA: Preferred Reporting Items for Systematic Reviews and Meta-Analyses
RCT: randomized controlled trial
SMD: standardized mean difference
