# Supplementary material for: Lifestyle eHealth and mHealth Interventions for Children and Adolescents: Systematic Umbrella Review and Meta–Meta-Analysis
Source: J Med Internet Res. 2025 Oct 17;27:e69065. doi: 10.2196/69065 (PMC12579299; doi:10.2196/69065)
Supplement: Multimedia Appendix 2 [file jmir_v27i1e69065_app2.docx]

# Supplementary Material 1. Database search strategy.

| MEDLINE(R) ALL <1946 to 2024>  Ovid MEDLINE(R) ALL <1946 to 2024> | |
| --- | --- |
| # Meta-Analysis and Systematic Review  1 Meta-Analysis as Topic/  2 meta analy$.tw.  3 metaanaly$.tw.  4 Meta-Analysis/  5 Systematic Review/  6 Systematic Reviews as Topic/  7 (systematic adj (review$1 or overview$1)).tw.  8 exp Review Literature as Topic/  9 or/1-8  # Databases  10 cochrane.ab.  11 embase.ab.  12 (psychlit or psyclit).ab.  13 (psychinfo or psycinfo).ab.  14 (cinahl or cinhal).ab.  15 science citation index.ab.  16 bids.ab.  17 cancerlit.ab.  18 or/10-17  # Search methods  19 reference list$.ab.  20 bibliograph$.ab.  21 hand-search$.ab.  22 relevant journals.ab.  23 manual search$.ab.  24 or/19-23  # Data extraction  25 selection criteria.ab.  26 data extraction.ab.  27 25 or 26  28 Review/  29 27 and 28  # Exclusions  30 Comment/  31 Letter/  32 Editorial/  33 animal/  34 human/  35 33 not (33 and 34)  36 or/30-32,35  # Combine searches  37 9 or 18 or 24 or 29  38 37 not 36 | # Physical activity and exercise  39 exp exercise/  40 exp exercise therapy/  41 exp sports/  42 Physical Fitness/  43 (physical* adj5 (fit* or train* or activ* or endur* or exer*)).ti,ab.  44 (exercis* adj5 (train* or physical* or activ*)).ti,ab.  45 sport*.ti,ab.  46 walk*.ti,ab.  47 swim*.ti,ab.  48 pilates.ti,ab.  49 step*.ti,ab.  50 HIIT.ti,ab.  51 (tai ji or tai chi or tai-ji or tai-chi).ti,ab.  52 (resistance adj3 train*).ti,ab.  # Sedentary behavior and screen time  53 exp Sedentary Behavior/  54 sedentary.ti,ab.  55 (sitting adj3 (time or hour* or minute*)).ti,ab.  56 screen time.ti,ab.  57 (television or TV or computer or smartphone or tablet).ti,ab.  58 (video adj2 (game* or gaming)).ti,ab.  59 ((recreational or leisure) adj2 (screen or media)).ti,ab.  # Weight management  60 exp Body Weight/  61 exp Body Weight Changes/  62 exp Weight Loss/  63 exp Weight Gain/  64 exp Obesity/  65 (weight adj3 (loss or losing or lost or reduc* or decreas* or watch* or control* or manage*)).ti,ab.  66 (weight adj3 (gain* or increas*)).ti,ab.  67 (overweight or over-weight or obes*).ti,ab.  68 (BMI or body mass index).ti,ab.  # Limited diet focus  69 (diet* adj3 (modif* or intervention* or change*)).ti,ab.  70 (calorie adj2 (restrict* or reduc*)).ti,ab.  # Combine all terms  71 or/39-70  # Final combination  72 38 and 71 |

# Supplementary Material 2. List of reasons on exclusion for all full texts (n=12).

|  | **Title** | **First author, year** | **Journal** | **Reason** |
| --- | --- | --- | --- | --- |
| 1 | Technology-based parenting interventions for children's physical and psychological health: a systematic review and meta-analysis | Flujas-Contreras, 2019 | Psychological Medicine | Adult population |
| 2 | Parent-Focused Childhood and Adolescent Overweight and Obesity eHealth Interventions: A Systematic Review and Meta-Analysis | Hammersley 2016 | Journal of Medical Internet Research | Adult population |
| 3 | Assessing the pragmatic nature of Mobile health interventions promoting physical activity: systematic review and meta-analysis | Stecher 2023 | JMIR mHealth and uHealth | Adult population |
| 4 | Assessing the effect of mHealth on child feeding practice in African countries: systematic and meta-analysis | Gilano 2023 | Journal of Health, Population and Nutrition | Adult population |
| 5 | Fitbit-based interventions for healthy lifestyle outcomes: systematic review and meta-analysis | Ringeval 2020 | Journal of Medical Internet Research | Adult population |
| 6 | Efficacy of mobile health applications to improve physical activity and sedentary behaviour: a systematic review and meta-analysis for physically inactive individuals | Zhang 2022 | International Journal of Environmental Research and Public Health | Adult population |
| 7 | The Effects of Active Video Games on Health-Related Physical Fitness and Motor Competence in Children and Adolescents with Healthy Weight: A Systematic Review and Meta-Analysis | Comeras-Chueca 2021 | International Journal of Environmental Research and Public Health | outcomes |
| 8 | Realizing the Effectiveness of Digital Interventions on Sedentary Behaviour (Physical Inactivity, Unhealthy Habit, Improper Diet) Monitoring and Prevention Approaches as a Meta-Analysis | Chatterjee | Journal of Medical Internet Research | Wrong outcomes |
| 9 | Effectiveness of Online and Remote Interventions for Mental Health in Children, Adolescents, and Young Adults After the Onset of the COVID-19 Pandemic: Systematic Review and Meta-Analysis | Fischer-Grote 2024 | JMIR Mental Health | Wrong outcomes |
| 10 | E-&mHealth interventions targeting nutrition, physical activity, sedentary behaviour, and/or obesity among children: A scoping review of systematic reviews and meta-analyses | Kracht 2021 | Obesity Reviews | Wrong study design |
| 11 | Effects of Consumer-Wearable Activity Tracker-Based Programs on Objectively Measured Daily Physical Activity and Sedentary Behavior Among School-Aged Children: A Systematic Review and Meta-analysis | Casado-Robles 2022 | Sports Medicine - Open | Wrong study design |
| 12 | Harnessing technological solutions for childhood obesity prevention and treatment: a systematic review and meta-analysis of current applications | Fowler 2021 | International Journal of Obesity | Wrong intervention |

References

1. Flujas-Contreras C, García-Palacios A, Gómez I. Technology-based parenting interventions for children's physical and psychological health: a systematic review and meta-analysis. Psychol Med. 2019;49(11):1787-98. doi:10.1017/S0033291719000349
2. Hammersley ML, Jones RA, Okely AD. Parent-focused childhood and adolescent overweight and obesity eHealth interventions: a systematic review and meta-analysis. J Med Internet Res. 2016;18(7):e203. doi:10.2196/jmir.5893
3. Stecher V, Kuhlmann T, Brunner C, et al. Assessing the pragmatic nature of mobile health interventions promoting physical activity: systematic review and meta-analysis. JMIR Mhealth Uhealth. 2023;11:e45121. doi:10.2196/45121
4. Gilano G, Getu M, Tsegaye G. Assessing the effect of mHealth on child feeding practice in African countries: systematic and meta-analysis. J Health Popul Nutr. 2023;42(1):47. doi:10.1186/s41043-023-00474-2
5. Ringeval M, Wagner G, Denford J, Paré G, Kitsiou S. Fitbit-based interventions for healthy lifestyle outcomes: systematic review and meta-analysis. J Med Internet Res. 2020;22(10):e23954. doi:10.2196/23954
6. Zhang Y, Li J, Guo S, Xu J, Qiu Y. Efficacy of mobile health applications to improve physical activity and sedentary behaviour: a systematic review and meta-analysis for physically inactive individuals. Int J Environ Res Public Health. 2022;19(19):12495. doi:10.3390/ijerph191912495
7. Comeras-Chueca C, Villalba-Heredia L, Pérez-Lasierra JL, Lozano-Berges G, Marín-Puyalto J, Vicente-Rodríguez G, et al. The effects of active video games on health-related physical fitness and motor competence in children and adolescents with healthy weight: a systematic review and meta-analysis. Int J Environ Res Public Health. 2021;18(24):12882. doi:10.3390/ijerph182412882
8. Chatterjee A, Prinz A, Gerdes M, Martinez S. Realizing the effectiveness of digital interventions on sedentary behaviour (physical inactivity, unhealthy habit, improper diet) monitoring and prevention approaches as a meta-analysis. J Med Internet Res. 2021;23(10):e26130. doi:10.2196/26130
9. Fischer-Grote L, Schultze-Lutter F, Voge J, Griebler U, Watzke B. Effectiveness of online and remote interventions for mental health in children, adolescents, and young adults after the onset of the COVID-19 pandemic: systematic review and meta-analysis. JMIR Ment Health. 2024;11:e49900. doi:10.2196/49900
10. Kracht CL, Webster EK, Staiano AE. E- and mHealth interventions targeting nutrition, physical activity, sedentary behaviour, and/or obesity among children: a scoping review of systematic reviews and meta-analyses. Obes Rev. 2021;22(11):e13316. doi:10.1111/obr.13316
11. Casado-Robles C, Heredia-León A, García-Hermoso A, et al. Effects of consumer-wearable activity tracker–based programs on objectively measured daily physical activity and sedentary behavior among school-aged children: a systematic review and meta-analysis. Sports Med Open. 2022;8(1):157. doi:10.1186/s40798-022-00527-6
12. Fowler LA, Grammer AC, Staiano AE. Harnessing technological solutions for childhood obesity prevention and treatment: a systematic review and meta-analysis of current applications. Int J Obes. 2021;45(9): 1679-91. doi:10.1038/s41366-021-00871-2

# Supplementary Material 3. Overview of all included systematic reviews and meta-analyses.

| **Author, year** | **No. of studies** | **Total sample** | **Population or condition**  **Gender**  **Age mean (SD) or range (years)** | **e-health and m-health intervention details** | **Outcomes of interest** |
| --- | --- | --- | --- | --- | --- |
| Ameryoun 2018 [49] | 10 | 1,402 | Overweight/obese children and adolescents  Females and males  Mean age range: 9.3 to 16 y | The interventions included various exergaming programs involving dance, active games, and sports, with sessions ranging from two to five times a week. They utilized equipment like dance pads, motion capture cameras, and cycling devices, promoting 40 to 130 minutes of moderate to vigorous physical activity per session. Additional elements included education on physical activity, nutritional behaviour, daily logs, team-building activities, and rewards for adherence, ensuring a comprehensive approach to enhancing physical fitness and reducing sedentary behaviour in children. Intervention range: 6 weeks to 1 year. | BMI |
| Azevedo 2022 [50] | 19 | 2,352 | Overweight/obese children and adolescents  Females and males  Mean age range: 5.4 to 15.7 y | The interventions involved various combinations of digital and face-to-face methods to support healthy lifestyle changes in adolescents. They included internet-based curricula with interactive sessions, goal setting, and SMS follow-ups, simplified lifestyle programs with cognitive behavioural components, use of wearable sensors, educational modules, psychoeducational telemedicine groups, gaming technology for physical activity, and motivational interviewing with self-monitoring and adaptive text messaging. Each intervention aimed to promote healthy eating, increase physical activity, and reduce sedentary behaviour, with several incorporating parental involvement and tailored communication strategies over periods ranging from six months to two years. Intervention range: 6 weeks to 12 months | BMI |
| Baumann 2022 [31] | 11 | 496 | Children and adolescents  Females and males  Mean age range: 3.5 to 17.8 y | The interventions include a variety of digital tools aimed at promoting physical activity and weight management. They encompass smartphone-based educational programs, web-based apps for behavioural support, SMS text messaging for daily behaviour feedback, and wearable devices like Fitbits for activity tracking. Some interventions also incorporate social components such as peer support groups on platforms like Facebook, game-based motivation strategies, and parent-focused online programs. Intervention range: 2-24 weeks | Insufficient physical activity  Sedentary behaviour |
| BeckSilva 2024 [32] | 13 | 9,603 | Adolescents  Females and males  Age range: 10 to 18 y | The interventions involved computer-based or internet-based nutrition education programs for adolescents, often providing personalized feedback based on questionnaires about dietary intake and physical activity habits. The programs typically included multiple sessions or modules delivered over weeks or months, drawing from theories like the Transtheoretical Model, Social Cognitive Theory, and the Theory of Planned Behaviour. Some interventions also involved components for parents like newsletters or counselling sessions to support behaviour change in the adolescents. Intervention range: 4 weeks to 2 years | Fat consumption  Fruits and vegetable consumption  BMI |
| Bossen 2020 [54] | 9 | 886 | Children with a chronic disease  Females and males  Mean age range: 9.9 to 15.7 y | Serious games: The interventions use various forms of technology to promote physical activity, combining educational content with interactive and engaging activities. They include web-based applications, exergames with consoles like PlayStation, Nintendo Wii, and Xbox, and tools such as cognitive behavioural strategies, goal setting, and feedback mechanisms to encourage participation and track progress. Interventions feature activities such as aerobic exercises, gross motor tasks, dance games, and interactive educational materials, often with incentiwves and positive reinforcement to motivate sustained engagement. Intervention range: 6-24 weeks | MVPA  Step counts  BMI |
| Bourke 2023 [51] | 12 | 721 | Overweight children and adolescents  Females and males  Age range: 7 to 18 y | The interventions primarily focus on incorporating active video games into children's routines to promote physical activity and weight management. They vary in duration, ranging from 6 to 24 weeks, and include a mix of home-based and supervised programs. Activities involve specific video games such as Dance Dance Revolution®, Nintendo® Wii, and Xbox Kinect, with structured schedules and progressive intensity, often integrated with broader weight management curricula and family involvement. Intervention range: 6-24 weeks | Weight  BMI |
| Butler 2022 [55] | 15 | 1438 | Children and young people living with juvenile idiopathic arthritis  Females and males  Age range: 4 to 18 y | The intervention involving a wearable activity tracker used the MisFit Flash to improve physical activity levels (PALs). Another intervention, Rheumates@Work, is a web-based program that employs behavioral and cognitive strategies to promote health. Both interventions leverage technology to encourage healthier lifestyles and improve physical activity among participants. Intervention range: 5 weeks – 3 months | Total physical activity  MVPA |
| Champion 2019 [33] | 22 | 18,873 | Adolescents  Females and males  Mean age: 13.4 y | eHealth (internet, computers, tablets, mobile technology, or tele-health). The interventions include a variety of educational formats such as computer-based education via CD-ROM and face-to-face lectures, tailored feedback, and interactive web-based and multimedia components. They incorporate individually tailored feedback based on behaviours and theoretical models, peer models in video sessions, healthy snack and exercise labs, and environmental changes in schools. Additional features across interventions include online discussion boards, expert advice, text messages, interactive learning websites, self-assessments, goal setting, and forums for peer and health coach interactions. Intervention range: 2 weeks to 36 months. | Fruit intake  Fruit and vegetable intake  Fat intake  Screen time  MVPA  Total physical activity |
| Comeras-Chueca 2021 [52] | 15 | 1554 | Children and adolescents with overweight or obesity  Females and males  Mean age range: 10.0 to 14 y | Exergames: The interventions involve various active video games (AVGs) like Nintendo Wii, Xbox Kinect, and PlayStation EyeToy, which use motion-sensing technology to encourage physical activity through immersive and interactive gameplay, including fitness-focused games (Wii Fit), competitive and cooperative play options, dance-based exergaming, and interactive cycling games (Gamebike). Intervention range: 6-24 weeks | BMI  Body fat percentage  Fat-free mass  Waist circumference |
| Darling 2017 [34] | 14 | 2369 | Children and adolescents  Females and males  Mean age range: 8.7 to 16 y | Exergames: The interventions focused on self-monitoring and behavioural changes through various technologies and methods. Several interventions utilized text messaging for self-monitoring of dietary intake and physical activity, sometimes paired with in-person sessions or smartphone applications to support behaviour tracking and provide feedback. Other strategies included incorporating game-based challenges, ecological momentary interventions, and family participation, emphasizing goal setting, tracking, and feedback to promote healthier behaviours, particularly increased fruit and vegetable consumption and reduced intake of unhealthy foods. Intervention range: 2-36 weeks | Paediatric weight status  Diet  Physical activity |
| Hernández-Jiménez 2019 [35] | 16 | 1272 | Children and adolescents  Females and males  Age range: 7 to 18 y | Active videogames: The interventions included various active videogame activities and programs aimed at improving physical activity and health among participants. Specific activities ranged from using GameBike and stationary cycling with music to using dance mats, Dance Revolution, Xbox 360, PlayStation Eye Toy USB, and Wii FitTM, with frequencies and durations varying from 1 to 3 times per week for 30 to 75 minutes. Some interventions also included additional components like nutrition education, behavioural management discussions, and family-centred didactics focusing on lifestyle changes. Intervention range: 10 to 16 weeks | BMI |
| He 2021 [36] | 9 | 558 | Children and Adolescents  Females and males  Mean age: 13.2 y | Smartphone interventions, including mobile exergames, monitoring with feedback and encouragement, goal-setting with encouragement, and immersive or non-immersive game apps, were utilized to promote physical activity. These interventions incorporated elements such as strengthening goals, action reminders, and encouragement to engage users in physical activity, demonstrating potential effectiveness in promoting PA through diverse mobile strategies. Intervention range: 2 weeks- 6 months | Total physical activity |
| Jiang 2024 [37] | 19 | 2971 | Preschool children  Females and males  Age range: 2 to 6 y | Various interventions aimed at promoting healthy behaviours in parents and young children include smartphone apps, motivational coaching, social media-based programs, text message interventions, web-based education, teacher-led programs, workshops, and gaming interventions. These interventions target behaviours such as physical activity, sedentary behaviour, and sleep through diverse delivery methods like technology-based platforms, coaching sessions, and educational materials. Intervention range: 1 week to 36 months | Total physical activity  MVPA  Sedentary time  Sleep |
| Lee 2016 [38] | 4 | 381 | Elementary school students  Females and males  Age: Not reported | Obesity or weight control using smartphone or mobile technology, including text messages. Intervention range: Not reported. | BMI  Daily exercise  Sugar beverage intake |
| Mazeas 2022 [39] | 16 | 2407 | Children, adolescents (and adults)  Females and males  Mean age range of children and adolescents: 10.3 to 17.8 y | The interventions comprised a mix of web-based, app-based, and exergame strategies aimed at promoting physical activity over various durations. These included gamification through the StepSmart Challenge, immersive storytelling in the Zombies, Run! 5K Training app, and synchronized mobile exergames like MobileKids Monster Manor, each with varying durations ranging from. Additionally, a mixed-reality conquering game app spanned 24 weeks, incentivizing both physical and social activities. Intervention range: 1 to 24 weeks | MVPA (adolescents only) |
| Oh 2022 [40] | 74 | 46,998 | Children and adolescents  Females and males  Mean age ranged: 7.7 to 18 y | The interventions included 19 studies using internet-based platforms, 5 using computer-based methods like CD-ROMs and programs, 8 utilizing text messaging or similar platforms, 5 developing or using mobile apps, and 9 employing active video games or exergames. Other interventions comprised 3 computer-based studies, 9 internet-based platform studies, 10 using active video games or exergames, 2 using messaging platforms, and 3 using mobile apps. Additionally, one study combined internet-based, mobile, and messaging platform elements: Intervention range: 2 weeks to 20 months. | Body fat  BMI  Screen time  Sedentary behaviour  MVPA  Fruit and vegetable intake |
| Oliveira 2020 [41] | 12 | 1016 | Children and adolescents  Females and males  Mean age range: 7 to 18 y | Exergames: The interventions involved exergames where participants used gaming consoles like Nintendo Wii, Playstation, and Xbox, playing games such as Dance Revolution, Just Dance, Wii Sports, and various sports and dance games. The duration and frequency of these interventions varied, including home-based and school-based settings, with sessions ranging from 30 to 60 minutes daily or multiple times weekly. Participants were often encouraged to substitute inactive video games with exergames and to meet physical activity recommendations, with some interventions incorporating parental involvement and step-count goals. Intervention range: 6 weeks to 10 months. | BMI  Physical activity levels |
| Park 2021 [42] | 10 | 640 | Childhood and adolescents  Females and males  Mean age range: 3.1 to 16.0 y | The interventions included various digital and telehealth strategies to promote healthy behaviors and physical activity. These encompassed displaying weight loss targets, exchanging motivational text messages, and using activity monitors to boost physical activity, along with semi structured telehealth dietitian consultations, informational websites, and supportive Facebook groups. Additional methods involved web-based exercise programs, apps for tracking energy intake and expenditure, personalized SMS feedback, internet-based curricula on nutrition and physical activity, and goal setting with feedback to improve behaviours and monitor progress in physical activity and diet. Intervention range: | BMI |
| Qiu 2022 [43] | 40 | 6,403 | Children and adolescents  Females and males  Mean age range: 7.5 to 16.1 y | eHealth (e.g., internet, computers, tablets, telehealth, mobile applications, phone calls, text messages, and emails) for delivering lifestyle interventions (e.g., dietary changes, physical activity, or behavioural therapy for weight management, such as self-monitoring, goal setting, or providing feedback) Intervention range: 6 weeks to 24 months. | BMI  Waist circumference  Body weight  Body fat |
| Sequí-Domínguez 2024 [44] | 20 | 6624 | Childhood and adolescents  Females and males  Age range: 8.4 to 17 y | eHealth: The interventions involved mobile apps, exergames, multicomponent interventions, telephone-delivered interventions, text messages, and web-based interventions for promoting physical activity. Most interventions were based on self-determination and social cognitive theories and typically included elements like coaching, counselling, and group sessions. The interventions often required clinicians' assistance, and some were autonomous, or school based. Intervention range: 8 to 96 weeks. | Activity counts  Steps per day  MVPA  LPA  Screen time  Sedentary time |
| Suleiman-Martos 2021 [45] | 23 | 11,280 | Childhood and adolescents  Females and males  Age range: 3 to 18 y | The interventions used various types of media, including nutrition board games, gain- and loss-framed nutrition video messages, and educational websites featuring serious games. Video games were a significant component, with multiple sessions designed to engage children in healthy eating habits. Other media included mobile applications, advergames, multimedia games, interactive modules, and educational apps, with session lengths ranging from a few minutes to over an hour. Intervention range: 5 days to 1 year | Nutritional knowledge  BMI (z-score) |
| Wang 2024 [46] | 28 | 5643 | Children and adolescents  Females and males  Mean age range: 2.5 to 14.9 y | The interventions included various combinations of mobile apps, wearable devices, and face-to-face sessions. Some groups used immersive or non-immersive fitness apps like "Zombies Run" and "Get Running", while others incorporated structured physical activity, nutritional counselling, and behaviour change programs supported by apps like "Fitbit", "MINISTOP", and "MapMyFitness". Additionally, social media, peer-based support, and regular feedback mechanisms such as SMS messages and clinical care enhancements were integrated to promote physical activity and healthy behaviours. Intervention range: 2-36 weeks. | Total physical activity  Sedentary behaviour  MVPA  BMI  Waist circumference |
| Wang 2022 [47] | 12 | 3227 | Childhood and adolescents  Females and males  Mean age: 13.2 y | The interventions involved using wearable devices like pedometers, wristbands, and Fitbits to track and encourage physical activity among children and adolescents. Some interventions set step count goals, provided feedback on activity levels, or offered rewards for meeting activity targets. Additionally, some interventions combined the use of wearable devices with educational components, such as online programs or text messaging interventions. Intervention range: 2.5-18 months. | BMI Body weight  Waist circumference  Body fat percentage |
| Yien 2021 [48] | 9 | 1,202 | Adolescents and pre-teens  Females and males  Age range: 8 to 18 y | The interventions involved using wearable sensors and wristbands for healthy lifestyle behaviour tracking and physical activity self-monitoring, along with pedometers for similar purposes over varying durations. Participants received online educational modules, text messages, social support via Facebook, apps, email, and phone calls, as well as telephonic coaching and communication through mobile and landline phones. Smartphone and tablet apps were utilized for real-time goal setting, self-monitoring, screen-time reduction, high energy intake management, and providing tips and feedback, with additional tools like tailored messaging and peer assessment to support physical activity and goal attainment. Intervention range: 3-24 months | Weight control |
| Zhu 2022 [53] | 8 | 3185 | Infants and children (included parents)  Females and males  Age range: 0 to 36 y | The interventions targeting sleep improvement utilize various technologies, including websites, DVDs, telephone calls, Facebook, emails, and text messages, either individually or in combination. These approaches leverage digital platforms and communication tools to provide information, support, and reminders aimed at enhancing sleep habits and quality.  Intervention range: 3 weeks to 11 months | Total sleep time  Wake after sleep onset  Sleep efficiency  Sleep onset latency |
| BMI: Body mass index; MVPA: moderate-to-vigorous physical activity; LPA: leisure time physical activity. | | | | | |

References

1. Ameryoun A, Sanaeinasab H, Saffari M, Koenig HG. Impact of Game-Based Health Promotion Programs on Body Mass Index in Overweight/Obese Children and Adolescents: A Systematic Review and Meta-Analysis of Randomized Controlled Trials. Child Obes. 2018 Feb/Mar;14(2):67-80.
2. Azevedo LB, Stephenson J, Ells L, et al. The effectiveness of e‐health interventions for the treatment of overweight or obesity in children and adolescents: A systematic review and meta‐analysis. Obesity Reviews 2022;23(2):e13373
3. Baumann H, Fiedler J, Wunsch K, Woll A, Wollesen B. mHealth interventions to reduce physical inactivity and sedentary behavior in children and adolescents: systematic review and meta-analysis of randomized controlled trials. JMIR mHealth and uHealth 2022;10(5):e35920.
4. Beck Silva KB, Miranda Pereira E, Santana MLPd, Costa PRF, Silva RdCR. Effects of computer-based interventions on food consumption and anthropometric parameters of adolescents: A systematic review and metanalysis. Critical Reviews in Food Science and Nutrition 2024;64(6):1617-1631.
5. Bossen D, Broekema A, Visser B, et al. Effectiveness of serious games to increase physical activity in children with a chronic disease: systematic review with meta-analysis. Journal Of Medical Internet Research 2020;22(4):e14549
6. Bourke M, Patterson L, Di Nardo F, Whittaker P, Verma A. Active video games and weight management in overweight children and adolescents—systematic review and meta-analysis. Journal of Public Health 2023;45(4):935-946.
7. Butler S, Sculley D, Santos D, et al. Effectiveness of eHealth and mHealth interventions supporting children and young people living with juvenile idiopathic arthritis: systematic review and meta-analysis. Journal of Medical Internet Research 2022;24(2):e30457.
8. Champion KE, Parmenter B, McGowan C, et al. Effectiveness of school-based eHealth interventions to prevent multiple lifestyle risk behaviours among adolescents: a systematic review and meta-analysis. The Lancet Digital Health 2019, Sep 1;5:e206-e221.
9. Comeras-Chueca C, Marin-Puyalto J, Matute-Llorente A, Vicente-Rodriguez G, Casajus JA, Gonzalez-Aguero A. Effects of active video games on health-related physical fitness and motor competence in children and adolescents with overweight or obesity: systematic review and meta-analysis. JMIR Serious Games 2021;9(4):e29981.
10. Darling KE, Sato AF. Systematic review and meta-analysis examining the effectiveness of mobile health technologies in using self-monitoring for pediatric weight management. Childhood Obesity 2017;13(5):347-355.
11. Hernández-Jiménez C, Sarabia R, Paz-Zulueta M, et al. Impact of active video games on body mass index in children and adolescents: systematic review and meta-analysis evaluating the quality of primary studies. International Journal Of Environmental Research And Public Health 2019;16(13):2424.
12. He Z, Wu H, Yu F, et al. Effects of smartphone-based interventions on physical activity in children and adolescents: systematic review and meta-analysis. JMIR mHealth and uHealth 2021;9(2):e22601.
13. Jiang S, Ng JY, Chong KH, Peng B, Ha AS. Effects of eHealth Interventions on 24-Hour Movement Behaviors Among Preschoolers: Systematic Review and Meta-Analysis. Journal of Medical Internet Research 2024, Feb 21;26:e52905.
14. Lee J, Piao M, Byun A, Kim J. A systematic review and meta-analysis of intervention for pediatric obesity using mobile technology. Nursing Informatics 2016;225:491-494.
15. Mazeas A, Duclos M, Pereira B, Chalabaev A. Evaluating the effectiveness of gamification on physical activity: systematic review and meta-analysis of randomized controlled trials. Journal Of Medical Internet Research 2022;24(1):e26779.
16. Oh C, Carducci B, Vaivada T, Bhutta ZA. Digital interventions for universal health promotion in children and adolescents: a systematic review. Pediatrics 2022;149 (Supp 5):e2021053852H.
17. Oliveira CB, Pinto RZ, Saraiva BT, et al. Effects of active video games on children and adolescents: A systematic review with meta‐analysis. Scandinavian Journal Of Medicine & Science In Sports 2020;30(1):4-12.
18. Park J, Park M-J, Seo Y-G. Effectiveness of information and communication technology on obesity in childhood and adolescence: systematic review and meta-analysis. Journal of Medical Internet Research 2021;23(11):e29003.
19. Qiu L-T, Sun G-X, Li L, Zhang J-D, Wang D, Fan B-Y. Effectiveness of multiple eHealth-delivered lifestyle strategies for preventing or intervening overweight/obesity among children and adolescents: A systematic review and meta-analysis. Frontiers in Endocrinology 2022, Sep 5;13:999702.
20. Sequí-Domínguez I, Cavero-Redondo I, Álvarez-Bueno C, López-Gil JF, Martínez-Vizcaíno V, Pascual-Morena C. Effectiveness of eHealth Interventions Promoting Physical Activity in Children and Adolescents: Systematic Review and Meta-Analysis. Journal of Medical Internet Research 2024, Feb 21; 26:e41649.
21. Suleiman-Martos N, García-Lara RA, Martos-Cabrera MB, et al. Gamification for the improvement of diet, nutritional habits, and body composition in children and adolescents: a systematic review and meta-analysis. Nutrients 2021;13(7):2478.
22. Wang J-W, Zhu Z, Shuling Z, et al. Effectiveness of mHealth App–Based Interventions for Increasing Physical Activity and Improving Physical Fitness in Children and Adolescents: Systematic Review and Meta-Analysis. JMIR mHealth and uHealth 2024;30(12):e51478.
23. Wang W, Cheng J, Song W, Shen Y. The effectiveness of wearable devices as physical activity interventions for preventing and treating obesity in children and adolescents: systematic review and meta-analysis. JMIR mHealth and uHealth 2022;10(4):e32435.
24. Yien J-M, Wang H-H, Wang R-H, Chou F-H, Chen K-H, Tsai F-S. Effect of mobile health technology on weight control in adolescents and preteens: a systematic review and meta-analysis. Frontiers in Public Health 2021;15(9):708321.
25. Zhu H, Xiao L, Tu A. Effectiveness of technology-based interventions for improving sleep among children: a systematic review and meta-analysis. Sleep Medicine 2022 Mar;91:141-150.

# Supplementary Material 4. AMSTAR-2 risk of bias and study quality ratings.

| Author, year | 1 | 2 | 3 | 4 | 5 | 6 | 7 | 8 | 9 | 10 | 11 | 12 | 13 | 14 | 15 | 16 | Overall confidence score |
| --- | --- | --- | --- | --- | --- | --- | --- | --- | --- | --- | --- | --- | --- | --- | --- | --- | --- |
| Ameryoun 2018 [49] | Y | N | Y | PY | N | Y | N | Y | Y | N | Y | N | N | Y | Y | Y | Critically low |
| Azevedo 2022 [50] | Y | Y | Y | PY | Y | Y | N | Y | Y | N | Y | Y | Y | Y | Y | Y | Low |
| Baumann 2022 [31] | Y | Y | Y | PY | Y | Y | N | Y | Y | N | Y | Y | Y | Y | Y | Y | Low |
| BeckSilva 2024 [32] | Y | Y | Y | Y | Y | Y | N | N | Y | N | Y | N | Y | Y | Y | Y | Low |
| Bossen 2020 [54] | Y | Y | Y | Y | Y | N | N | Y | Y | N | Y | Y | Y | Y | N | Y | Critically low |
| Bourke 2023 [51] | Y | N | Y | PY | Y | Y | N | Y | Y | N | Y | N | N | Y | N | Y | Critically low |
| Butler 2022 [55] | Y | Y | Y | PY | Y | Y | N | Y | Y | N | Y | Y | N | Y | N | Y | Critically low |
| Champion 2019 [33] | Y | Y | Y | Y | Y | Y | Y | Y | Y | N | Y | N | Y | Y | Y | Y | Moderate |
| Comeras-Chueca 2021 [52] | Y | Y | Y | PY | Y | N | N | N | Y | N | Y | N | N | Y | N | Y | Critically low |
| Darling 2017 [34] | N | Y | Y | PY | N | N | N | N | Y | N | Y | N | N | N | N | Y | Critically low |
| Hernández-Jiménez 2019 [35] | Y | N | Y | PY | N | Y | N | Y | Y | N | Y | Y | N | Y | Y | Y | Critically low |
| He 2021 [36] | Y | Y | Y | Y | Y | Y | N | PY | Y | N | Y | Y | N | Y | Y | Y | Critically low |
| Jiang 2024 [37] | Y | Y | Y | PY | Y | Y | N | PY | Y | N | Y | Y | Y | Y | Y | Y | Low |
| Lee 2016 [38] | N | N | Y | PY | Y | Y | N | N | N | N | Y | N | N | N | N | N | Critically low |
| Mazeas 2022 [39] | Y | Y | Y | Y | Y | Y | N | N | Y | N | Y | Y | Y | Y | Y | Y | Low |
| Oh 2022 [40] | Y | Y | Y | PY | Y | Y | N | Y | Y | N | Y | N | Y | Y | N | Y | Critically low |
| Oliveira 2020 [41] | Y | Y | Y | Y | Y | Y | Y | PY | Y | N | Y | Y | N | Y | N | Y | Critically low |
| Park 2021 [42] | Y | N | Y | PY | Y | Y | N | Y | Y | N | Y | Y | N | Y | Y | Y | Critically low |
| Qiu 2022 [43] | Y | N | Y | PY | Y | Y | N | N | Y | N | Y | N | N | Y | Y | Y | Critically low |
| Sequí-Domínguez 2024 [44] | Y | Y | Y | PY | Y | Y | Y | PY | Y | N | Y | Y | Y | Y | Y | Y | Moderate |
| Suleiman-Martos 2021 [45] | Y | N | Y | PY | Y | Y | N | N | Y | N | Y | N | Y | Y | Y | Y | Critically low |
| Wang 2024 [46] | Y | Y | Y | PY | Y | Y | N | Y | Y | N | Y | N | N | Y | Y | Y | Critically low |
| Wang 2022 [47] | Y | N | Y | PY | Y | Y | N | PY | Y | N | Y | N | Y | Y | N | Y | Critically low |
| Yien 2021 [48] | N | N | Y | PY | N | Y | N | N | Y | N | Y | N | N | N | Y | Y | Critically low |
| Zhu 2022 [53] | N | Y | Y | PY | Y | Y | N | N | Y | N | Y | N | N | Y | N | Y | Critically low |
| N: no; PY: partial yes; Y: yes.  AMSTAR-2 Items  1. Did the research questions and inclusion criteria for the review include the components of PICO?  2. Did the report of the review contain an explicit statement that the review methods were established prior to the conduct of the review and did the report justify any significant deviations from the protocol?  3. Did the review authors explain their selection of the study designs for inclusion in the review?  4. Did the review authors use a comprehensive literature search strategy?  5. Did the review authors perform study selection in duplicate?  6. Did the review authors perform data extraction in duplicate?  7. Did the review authors provide a list of excluded studies and justify the exclusions?  8. Did the review authors describe the included studies in adequate detail?  9. Did the review authors use a satisfactory technique for assessing the risk of bias (RoB) in individual studies that were included in the review?  10. Did the review authors report on the sources of funding for the studies included in the review?  11. If meta-analysis was performed did the review authors use appropriate methods for statistical combination of results?  12. If meta-analysis was performed, did the review authors assess the potential impact of RoB in individual studies on the results of the meta-analysis or other evidence synthesis?  13. Did the review authors account for RoB in individual studies when interpreting/ discussing the results of the review?  14. Did the review authors provide a satisfactory explanation for, and discussion of, any heterogeneity observed in the results of the review?  15. If they performed quantitative synthesis did the review authors carry out an adequate investigation of publication bias (small study bias) and discuss its likely impact on the results of the review?  16. Did the review authors report any potential sources of conflict of interest, including any funding they received for conducting the review? | | | | | | | | | | | | | | | | | |

References

1. Ameryoun A, Sanaeinasab H, Saffari M, Koenig HG. Impact of Game-Based Health Promotion Programs on Body Mass Index in Overweight/Obese Children and Adolescents: A Systematic Review and Meta-Analysis of Randomized Controlled Trials. Child Obes. 2018 Feb/Mar;14(2):67-80.
2. Azevedo LB, Stephenson J, Ells L, et al. The effectiveness of e‐health interventions for the treatment of overweight or obesity in children and adolescents: A systematic review and meta‐analysis. Obesity Reviews 2022;23(2):e13373
3. Baumann H, Fiedler J, Wunsch K, Woll A, Wollesen B. mHealth interventions to reduce physical inactivity and sedentary behavior in children and adolescents: systematic review and meta-analysis of randomized controlled trials. JMIR mHealth and uHealth 2022;10(5):e35920.
4. Beck Silva KB, Miranda Pereira E, Santana MLPd, Costa PRF, Silva RdCR. Effects of computer-based interventions on food consumption and anthropometric parameters of adolescents: A systematic review and metanalysis. Critical Reviews in Food Science and Nutrition 2024;64(6):1617-1631.
5. Bossen D, Broekema A, Visser B, et al. Effectiveness of serious games to increase physical activity in children with a chronic disease: systematic review with meta-analysis. Journal Of Medical Internet Research 2020;22(4):e14549
6. Bourke M, Patterson L, Di Nardo F, Whittaker P, Verma A. Active video games and weight management in overweight children and adolescents—systematic review and meta-analysis. Journal of Public Health 2023;45(4):935-946.
7. Butler S, Sculley D, Santos D, et al. Effectiveness of eHealth and mHealth interventions supporting children and young people living with juvenile idiopathic arthritis: systematic review and meta-analysis. Journal of Medical Internet Research 2022;24(2):e30457.
8. Champion KE, Parmenter B, McGowan C, et al. Effectiveness of school-based eHealth interventions to prevent multiple lifestyle risk behaviours among adolescents: a systematic review and meta-analysis. The Lancet Digital Health 2019, Sep 1;5:e206-e221.
9. Comeras-Chueca C, Marin-Puyalto J, Matute-Llorente A, Vicente-Rodriguez G, Casajus JA, Gonzalez-Aguero A. Effects of active video games on health-related physical fitness and motor competence in children and adolescents with overweight or obesity: systematic review and meta-analysis. JMIR Serious Games 2021;9(4):e29981.
10. Darling KE, Sato AF. Systematic review and meta-analysis examining the effectiveness of mobile health technologies in using self-monitoring for pediatric weight management. Childhood Obesity 2017;13(5):347-355.
11. Hernández-Jiménez C, Sarabia R, Paz-Zulueta M, et al. Impact of active video games on body mass index in children and adolescents: systematic review and meta-analysis evaluating the quality of primary studies. International Journal Of Environmental Research And Public Health 2019;16(13):2424.
12. He Z, Wu H, Yu F, et al. Effects of smartphone-based interventions on physical activity in children and adolescents: systematic review and meta-analysis. JMIR mHealth and uHealth 2021;9(2):e22601.
13. Jiang S, Ng JY, Chong KH, Peng B, Ha AS. Effects of eHealth Interventions on 24-Hour Movement Behaviors Among Preschoolers: Systematic Review and Meta-Analysis. Journal of Medical Internet Research 2024, Feb 21;26:e52905.
14. Lee J, Piao M, Byun A, Kim J. A systematic review and meta-analysis of intervention for pediatric obesity using mobile technology. Nursing Informatics 2016;225:491-494.
15. Mazeas A, Duclos M, Pereira B, Chalabaev A. Evaluating the effectiveness of gamification on physical activity: systematic review and meta-analysis of randomized controlled trials. Journal Of Medical Internet Research 2022;24(1):e26779.
16. Oh C, Carducci B, Vaivada T, Bhutta ZA. Digital interventions for universal health promotion in children and adolescents: a systematic review. Pediatrics 2022;149 (Supp 5):e2021053852H.
17. Oliveira CB, Pinto RZ, Saraiva BT, et al. Effects of active video games on children and adolescents: A systematic review with meta‐analysis. Scandinavian Journal Of Medicine & Science In Sports 2020;30(1):4-12.
18. Park J, Park M-J, Seo Y-G. Effectiveness of information and communication technology on obesity in childhood and adolescence: systematic review and meta-analysis. Journal of Medical Internet Research 2021;23(11):e29003.
19. Qiu L-T, Sun G-X, Li L, Zhang J-D, Wang D, Fan B-Y. Effectiveness of multiple eHealth-delivered lifestyle strategies for preventing or intervening overweight/obesity among children and adolescents: A systematic review and meta-analysis. Frontiers in Endocrinology 2022, Sep 5;13:999702.
20. Sequí-Domínguez I, Cavero-Redondo I, Álvarez-Bueno C, López-Gil JF, Martínez-Vizcaíno V, Pascual-Morena C. Effectiveness of eHealth Interventions Promoting Physical Activity in Children and Adolescents: Systematic Review and Meta-Analysis. Journal of Medical Internet Research 2024, Feb 21; 26:e41649.
21. Suleiman-Martos N, García-Lara RA, Martos-Cabrera MB, et al. Gamification for the improvement of diet, nutritional habits, and body composition in children and adolescents: a systematic review and meta-analysis. Nutrients 2021;13(7):2478.
22. Wang J-W, Zhu Z, Shuling Z, et al. Effectiveness of mHealth App–Based Interventions for Increasing Physical Activity and Improving Physical Fitness in Children and Adolescents: Systematic Review and Meta-Analysis. JMIR mHealth and uHealth 2024;30(12):e51478.
23. Wang W, Cheng J, Song W, Shen Y. The effectiveness of wearable devices as physical activity interventions for preventing and treating obesity in children and adolescents: systematic review and meta-analysis. JMIR mHealth and uHealth 2022;10(4):e32435.
24. Yien J-M, Wang H-H, Wang R-H, Chou F-H, Chen K-H, Tsai F-S. Effect of mobile health technology on weight control in adolescents and preteens: a systematic review and meta-analysis. Frontiers in Public Health 2021;15(9):708321.
25. Zhu H, Xiao L, Tu A. Effectiveness of technology-based interventions for improving sleep among children: a systematic review and meta-analysis. Sleep Medicine 2022 Mar;91:141-150.

# Supplementary Material 5. Meta-analysis of effects of eHealth and mHealth interventions on MVPA in children and adolescents based on mean difference (MD).

Mean difference effect size = minutes/day

References

1. Sequí-Domínguez I, Cavero-Redondo I, Álvarez-Bueno C, López-Gil JF, Martínez-Vizcaíno V, Pascual-Morena C. Effectiveness of eHealth Interventions Promoting Physical Activity in Children and Adolescents: Systematic Review and Meta-Analysis. Journal of Medical Internet Research 2024, Feb 21; 26:e41649.
2. Oh C, Carducci B, Vaivada T, Bhutta ZA. Digital interventions for universal health promotion in children and adolescents: a systematic review. Pediatrics 2022;149 (Supp 5):e2021053852H.

# Supplementary Material 6. Meta-analysis of effects of eHealth and mHealth interventions on sedentary behaviour in children and adolescents based on mean difference (MD).

Mean difference = Minutes/day

References

1. Sequí-Domínguez I, Cavero-Redondo I, Álvarez-Bueno C, López-Gil JF, Martínez-Vizcaíno V, Pascual-Morena C. Effectiveness of eHealth Interventions Promoting Physical Activity in Children and Adolescents: Systematic Review and Meta-Analysis. Journal of Medical Internet Research 2024, Feb 21; 26:e41649.
2. Oh C, Carducci B, Vaivada T, Bhutta ZA. Digital interventions for universal health promotion in children and adolescents: a systematic review. Pediatrics 2022;149 (Supp 5):e2021053852H.

# Supplementary Material 7. Meta-analysis of effects of eHealth and mHealth interventions on screentime in children and adolescents based on mean difference (MD).

Mean difference = Minutes/day

References

1. Sequí-Domínguez I, Cavero-Redondo I, Álvarez-Bueno C, López-Gil JF, Martínez-Vizcaíno V, Pascual-Morena C. Effectiveness of eHealth Interventions Promoting Physical Activity in Children and Adolescents: Systematic Review and Meta-Analysis. Journal of Medical Internet Research 2024, Feb 21; 26:e41649.
2. Oh C, Carducci B, Vaivada T, Bhutta ZA. Digital interventions for universal health promotion in children and adolescents: a systematic review. Pediatrics 2022;149 (Supp 5):e2021053852H.

# Supplementary Material 8. Meta-analysis of effects of e- and m-health interventions on sleep duration in children and adolescents based on standardised mean difference (SMD). Positive effect sizes favour intervention.

References

1. Zhu H, Xiao L, Tu A. Effectiveness of technology-based interventions for improving sleep among children: a systematic review and meta-analysis. Sleep Medicine 2022 Mar;91:141-150.
2. Jiang S, Ng JY, Chong KH, Peng B, Ha AS. Effects of eHealth Interventions on 24-Hour Movement Behaviors Among Preschoolers: Systematic Review and Meta-Analysis. Journal of Medical Internet Research 2024, Feb 21;26:e52905.

# Supplementary Material 9. Meta-analysis of effects of eHealth and mHealth interventions on BMI in children and adolescents based on mean difference (MD).

Mean difference effect size = kg/m^2^

References

1. Bourke M, Patterson L, Di Nardo F, Whittaker P, Verma A. Active video games and weight management in overweight children and adolescents—systematic review and meta-analysis. Journal of Public Health 2023;45(4):935-946.
2. Park J, Park M-J, Seo Y-G. Effectiveness of information and communication technology on obesity in childhood and adolescence: systematic review and meta-analysis. Journal of Medical Internet Research 2021;23(11):e29003.
3. Qiu L-T, Sun G-X, Li L, Zhang J-D, Wang D, Fan B-Y. Effectiveness of multiple eHealth-delivered lifestyle strategies for preventing or intervening overweight/obesity among children and adolescents: A systematic review and meta-analysis. Frontiers in Endocrinology 2022, Sep 5;13:999702.
4. Wang J-W, Zhu Z, Shuling Z, et al. Effectiveness of mHealth App–Based Interventions for Increasing Physical Activity and Improving Physical Fitness in Children and Adolescents: Systematic Review and Meta-Analysis. JMIR mHealth and uHealth 2024;30(12):e51478.
5. Wang W, Cheng J, Song W, Shen Y. The effectiveness of wearable devices as physical activity interventions for preventing and treating obesity in children and adolescents: systematic review and meta-analysis. JMIR mHealth and uHealth 2022;10(4):e32435.
6. Comeras-Chueca C, Marin-Puyalto J, Matute-Llorente A, Vicente-Rodriguez G, Casajus JA, Gonzalez-Aguero A. Effects of active video games on health-related physical fitness and motor competence in children and adolescents with overweight or obesity: systematic review and meta-analysis. JMIR Serious Games 2021;9(4):e29981.
7. Beck Silva KB, Miranda Pereira E, Santana MLPd, Costa PRF, Silva RdCR. Effects of computer-based interventions on food consumption and anthropometric parameters of adolescents: A systematic review and metanalysis. Critical Reviews in Food Science and Nutrition 2024;64(6):1617-1631.

# Supplementary Material 10. Meta-analysis of effects of eHealth and mHealth interventions on bodyweight in children and adolescents based on mean difference (MD).

Mean difference effect size = kilograms

References

1. Qiu L-T, Sun G-X, Li L, Zhang J-D, Wang D, Fan B-Y. Effectiveness of multiple eHealth-delivered lifestyle strategies for preventing or intervening overweight/obesity among children and adolescents: A systematic review and meta-analysis. Frontiers in Endocrinology 2022, Sep 5;13:999702.
2. Wang W, Cheng J, Song W, Shen Y. The effectiveness of wearable devices as physical activity interventions for preventing and treating obesity in children and adolescents: systematic review and meta-analysis. JMIR mHealth and uHealth 2022;10(4):e32435.

# Supplementary Material 11. Meta-analysis of effects of eHealth and mHealth interventions on body fat in children and adolescents based on mean difference (MD).

Mean difference effect size = Percent body fat (%)

References

1. Wang W, Cheng J, Song W, Shen Y. The effectiveness of wearable devices as physical activity interventions for preventing and treating obesity in children and adolescents: systematic review and meta-analysis. JMIR mHealth and uHealth 2022;10(4):e32435.
2. Qiu L-T, Sun G-X, Li L, Zhang J-D, Wang D, Fan B-Y. Effectiveness of multiple eHealth-delivered lifestyle strategies for preventing or intervening overweight/obesity among children and adolescents: A systematic review and meta-analysis. Frontiers in Endocrinology 2022, Sep 5;13:999702.
3. Comeras-Chueca C, Marin-Puyalto J, Matute-Llorente A, Vicente-Rodriguez G, Casajus JA, Gonzalez-Aguero A. Effects of active video games on health-related physical fitness and motor competence in children and adolescents with overweight or obesity: systematic review and meta-analysis. JMIR Serious Games 2021;9(4):e29981.
4. Oh C, Carducci B, Vaivada T, Bhutta ZA. Digital interventions for universal health promotion in children and adolescents: a systematic review. Pediatrics 2022;149 (Supp 5):e2021053852H.

# Supplementary Material 12. Meta-analysis of effects of eHealth and mHealth interventions on waist circumference in children and adolescents based on mean difference (MD).

Mean difference effect size = centimetres

References

1. Qiu L-T, Sun G-X, Li L, Zhang J-D, Wang D, Fan B-Y. Effectiveness of multiple eHealth-delivered lifestyle strategies for preventing or intervening overweight/obesity among children and adolescents: A systematic review and meta-analysis. Frontiers in Endocrinology 2022, Sep 5;13:999702.
2. Comeras-Chueca C, Marin-Puyalto J, Matute-Llorente A, Vicente-Rodriguez G, Casajus JA, Gonzalez-Aguero A. Effects of active video games on health-related physical fitness and motor competence in children and adolescents with overweight or obesity: systematic review and meta-analysis. JMIR Serious Games 2021;9(4):e29981.
3. Wang J-W, Zhu Z, Shuling Z, et al. Effectiveness of mHealth App–Based Interventions for Increasing Physical Activity and Improving Physical Fitness in Children and Adolescents: Systematic Review and Meta-Analysis. JMIR mHealth and uHealth 2024;30(12):e51478.
4. Wang W, Cheng J, Song W, Shen Y. The effectiveness of wearable devices as physical activity interventions for preventing and treating obesity in children and adolescents: systematic review and meta-analysis. JMIR mHealth and uHealth 2022;10(4):e32435.

# Supplementary Material 13. Subgroup meta-analyses of moderate-to-vigorous physical activity

|  | **Number of effects** | **SMD** | **95% CI** | **I^2^ (%)** | **Test of subgroup differences** |
| --- | --- | --- | --- | --- | --- |
| **Age** |  |  |  |  | Q_b_ (1) = 0.02, p = 0.88 |
| <13 years | 4 | 0.23 | 0.09, 0.39 | 93.42 |  |
| ≥13 years | 3 | 0.25 | 0.14, 0.36 | 0 |  |
| **Intervention duration** |  |  |  |  | Q_b_ (1) = 9.03, p < 0.01 |
| <8 weeks | 2 | 0.86 | 0.44, 1.28 | 0 |  |
| ≥8 weeks | 3 | 0.19 | 0.07, 0.32 | 0 |  |
| **Intervention** |  |  |  |  | Q_b_ (7) = 7.08, p = 0.42 |
| E-health only (various) | 2 | 0.19 | 0.00, 0.38 | 35.73 |  |
| M-health only (various) | 4 | 0.32 | 0.18, 0.45 | 0 |  |
| Exergames | 3 | 0.23 | -0.04, 0.50 | 35.59 |  |
| E- and m-health mixed | 1 | 0.36 | -0.37, 1.09 | - |  |
| Web-based only | 1 | 0.04 | -0.43, 0.51 | - |  |
| App only | 1 | 0.76 | 0.23, 1.29 | - |  |
| SMS only | 1 | 0.18 | -0.06, 0.42 | - |  |
| SMS + app | 1 | -0.03 | -0.55, 0.49 | - |  |
| **Risk of bias score** |  |  |  |  | Q_b_ (2) = 1.06, p = 0.59 |
| Moderate | 1 | 0.33 | 0.05, 0.61 | - |  |
| Low | 2 | 0.17 | 0.04, 0.30 | 0 |  |
| Critically low | 3 | 0.18 | 0.00, 0.36 | 0 |  |

# Supplementary Material 14. Subgroup meta-analyses of total physical activity

|  | **Number of effects** | **SMD** | **95% CI** | **I^2^ (%)** | **Test of subgroup differences** |
| --- | --- | --- | --- | --- | --- |
| **Age** |  |  |  |  | Q_b_ (1) = 3.21, p = 0.38 |
| <13 years | 2 | 0.02 | -0.14, 0.17 | 54.72 |  |
| ≥13 years | 1 | 0.42 | 0.01, 0.83 | - |  |
| **Intervention duration** |  |  |  |  | Q_b_ (1) = 1.66, p = 0.20 |
| <8 weeks | 2 | 0.81 | -0.33, 1.96 | 93.07 |  |
| ≥8 weeks | 3 | 0.05 | -0.03, 0.14 | 0 |  |
| **Intervention** |  |  |  |  | Q_b_ (3) = 1.28, p = 0.73 |
| E-health (various) | 2 | 0.19 | 0.00, 0.38 | 35.73 |  |
| M-health (various) | 4 | 0.32 | 0.18, 0.45 | 0 |  |
| Exergames | 2 | 0.21 | -0.14, 0.57 | 58.45 |  |
| E- and m-health mixed | 1 | 0.36 | -0.37, 1.09 | - |  |
| **Risk of bias score** |  |  |  |  | Q_b_ (2) = 4.48, p = 0.11 |
| Moderate | 1 | 0.14 | 0.05, 0.23 | - |  |
| Low | 2 | 0.40 | 0.13, 0.66 | 0 |  |
| Critically low | 6 | 0.26 | 0.12, 0.40 | 13.01 |  |

# Supplementary Material 15. Subgroup meta-analyses of body mass index.

|  | **Number of effects** | **SMD** | **95% CI** | **I^2^ (%)** | **Test of subgroup differences** |
| --- | --- | --- | --- | --- | --- |
| **Age** |  |  |  |  | Q_b_ (1) = 0.35, p = 0.55 |
| <13 years | 5 | 0.26 | 0.16, 0.35 | 0 |  |
| ≥13 years | 3 | 0.34 | 0.08, 0.61 | 0 |  |
| **Intervention duration** |  |  |  |  | Q_b_ (1) = 6.08, p < 0.01 |
| <12 weeks | 3 | -0.07 | -0.35, 0.22 | 48.16 |  |
| ≥12 weeks | 3 | 0.46 | 0.15, 0.76 | 63.79 |  |
| **Session frequency** |  |  |  |  | Q_b_ (5) = 38.00, p < 0.01 |
| E-health (various) | 1 | 0.31 | 0.13, 0.49 | - |  |
| M-health (various) | 1 | 0.07 | -0.16, 0.31 | - |  |
| Exergames | 6 | 0.22 | 0.14, 0.29 | 0 |  |
| E- and m-health mixed | 2 | 0.20 | -0.08, 0.48 | 62.91 |  |
| Web-based only | 1 | 0.00 | -0.06, 0.06 | - |  |
| App only | 1 | 0.78 | 0.43, 1.13 | - |  |
| **Risk of bias score** |  |  |  |  | Q_b_ (1) = 2.09, p=0.15 |
| Low | 7 | 0.17 | 0.09, 0.24 | 34.25 |  |
| Critically low | 1 | 0.31 | 0.13, 0.49 | - |  |

# Supplementary Material 16. Subgroup meta-analyses of sedentary behaviour.

|  | **Number of effects** | **SMD** | **95% CI** | **I^2^ (%)** | **Test of subgroup differences** |
| --- | --- | --- | --- | --- | --- |
| **Age** |  |  |  |  | Q_b_ (1) = 2.25, p = 0.13 |
| <13 years | 2 | 2.36 | -0.44, 5.16 | 99.07 |  |
| ≥13 years | 1 | 0.21 | 0.00, 0.42 | - |  |
| **Intervention duration** |  |  |  |  | Q_b_ (1) = 0.21, p = 0.65 |
| <12 weeks | 2 | 0.41 | 0.13, 0.68 | 37.12 |  |
| ≥12 weeks | 2 | 0.71 | -0.55, 1.96 | 88.67 |  |
| **Intervention** |  |  |  |  | Q_b_ (2) = 16.2, p <0.01 |
| E-health (various) | 2 | 0.11 | 0.04, 0.17 | 0 |  |
| M-health (various) | 1 | -0.11 | -0.23, 0.01 | - |  |
| Wearables | 1 | 0.97 | 0.28, 1.67 | - |  |
| **Risk of bias score** |  |  |  |  | Q_b_ (2) = 6.44, p = 0.04 |
| Moderate | 1 | 0.09 | 0.01, 0.17 | - |  |
| Low | 2 | 0.02 | -0.24, 0.27 | 88.4% |  |
| Critically low | 1 | 0.97 | 0.28, 1.67 | - |  |

# Supplementary Material 17. Funnel plot for studies assessing physical activity outcomes.
